# Hunter-Gatherer Inter-Band Interaction Rates: Implications for Cumulative Culture

**DOI:** 10.1371/journal.pone.0102806

**Published:** 2014-07-21

**Authors:** Kim R. Hill, Brian M. Wood, Jacopo Baggio, A. Magdalena Hurtado, Robert T. Boyd

**Affiliations:** 1 School of Human Evolution and Social Change, Arizona State University, Tempe, Arizona, United States of America; 2 Department of Anthropology, Yale University, New Haven, Connecticut, United States of America; Bristol University, United Kingdom

## Abstract

Our species exhibits spectacular success due to cumulative culture. While cognitive evolution of social learning mechanisms may be partially responsible for adaptive human culture, features of early human social structure may also play a role by increasing the number potential models from which to learn innovations. We present interview data on interactions between same-sex adult dyads of Ache and Hadza hunter-gatherers living in multiple distinct residential bands (20 Ache bands; 42 Hadza bands; 1201 dyads) throughout a tribal home range. Results show high probabilities (5%–29% per year) of cultural and cooperative interactions between randomly chosen adults. Multiple regression suggests that ritual relationships increase interaction rates more than kinship, and that affinal kin interact more often than dyads with no relationship. These may be important features of human sociality. Finally, yearly interaction rates along with survival data allow us to estimate expected lifetime partners for a variety of social activities, and compare those to chimpanzees. Hadza and Ache men are estimated to observe over 300 men making tools in a lifetime, whereas male chimpanzees interact with only about 20 other males in a lifetime. High intergroup interaction rates in ancestral humans may have promoted the evolution of cumulative culture.

## Introduction

It has been hypothesized that cumulative culture and extensive non-kin cooperation allowed Homo sapiens to replace other hominin species in the Pleistocene and facilitated the biological dominance of our species in the Holocene [1]. In order to understand the emergence of these features we must examine aspects of social behavior in our ancestors that may have favored their evolution. Observations of modern hunter-gatherers offer the opportunity to examine features of that lifestyle that may be associated with important evolved human traits. Within-band interactions such as non-kin food sharing [2], cooperative food acquisition, and provisioning of multiple goods and services [3] are well documented for recent hunter-gatherers [4], [5] and part of a cooperative breeding life history that may be critical for explaining human success. But, between-band interactions may also be important for understanding the unique nature of our species. Inter-band social networks are hypothesized to explain evolved brain expansion [6], [7], extensive non-kin cooperation [8], [9], [10] and the emergence of cumulative culture [11], [12], [13].

Early ethnographers suggested that hunter-gatherer societies were primarily kin based [14], [15], and hence between-group interactions might be primarily associated with genetic kinship. More recently however, large interaction networks in our species are hypothesized to derive from pair bonding in ancestral hunter-gatherer societies, with recognition of affines producing a unique metaband (ie. tribal) social structure, not found in any other primate [16], [17]. Finally, cultural institutions such as ritualized partnerships and complex marriage rules have been hypothesized as features designed to promote between-band interaction in foragers [18], [19], [20]. In this paper we examine the effects of all three of these on interband interactions. While recent studies have examined reported preferential association networks [21], scientists still have no quantitative measure from any hunter-gatherer society of actual interaction rates between individuals residing in different bands. Here we present the first study designed to provide quantitative estimates of inter-band interaction rates and examine the impact of genetic and affinal kinship and ritual relationships on these rates.

## Methods

### Ethics statement

This study was reviewed and approved by the institutional Review Board of Arizona State University and Stanford University. Because informants were illiterate and spoke only native languages, general consent was obtained in community meetings and subsequent individual verbal consent was obtained from all participants as per IRB approval.

### Study populations

Ache hunter-gatherers roamed the forests of eastern Paraguay exploiting palm starch and hunting mammals with bow and arrow until pacification in the 1970s [22]. The Ache “tribe” is defined by a single mutually intelligible language and shared cultural features absent from the surrounding Guarani horticultural tribes (see Text S1, for details). The Ache had no pan-tribal political or religious leaders and the entire tribe never gathered together in one place for any political or religious functions. Within the Ache tribe, there were four regional “sub-tribes” in the 20^th^ century (Figure S1 in File S1), defined by dialect and minor cultural differences. Ache sub-tribes were further subdivided into multiple residential bands that camped together and formed the basis of daily living. Because of visiting and migration, band composition was stable over periods of months but not years.

**Figure 1:**
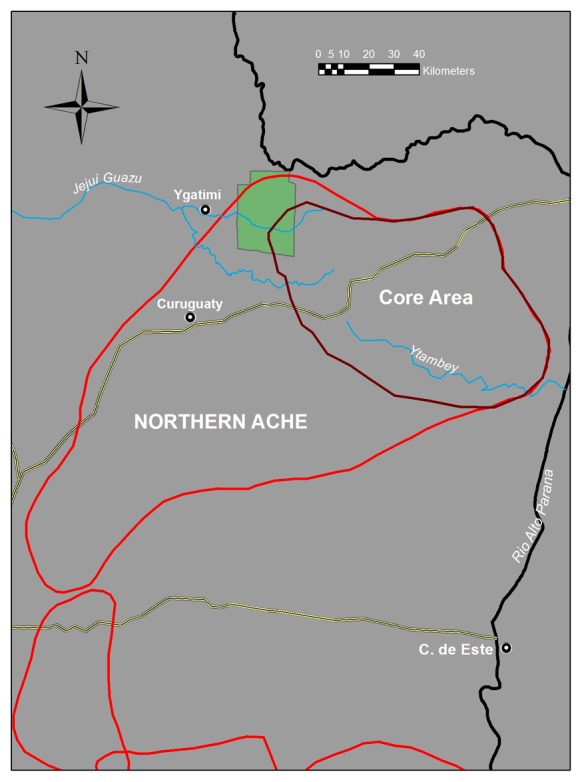
Northern Ache maximal boundaries and core use area during the second half of the 20th century. Bands were distributed throughout the core area, occassionally visiting more remote tribal home range in the Southwest. Band location moved almost daily with no restricted localities. The construction of the road passing through Curuguaty and the core Ache territualory was the impetus for permanent peaceful outside contact in the 1970s. The green square shows the modern day Mbarcayu reserve.

The Northern Ache were the largest Ache sub-tribe, consisting of about 560 people living in 18–20 residential bands during the last decade before peaceful outside contact [17]. Northern Ache bands were usually dispersed throughout their core home range of ∼5,270 km^2^ (Figure 1) and informants report that the different residential bands were generally located at distances of 10–30 km from each other. Occasionally these bands roamed as far as 150 km apart when they made temporary use of distant areas in their ∼14,630 km^2^ maximal home range.

**Figure 2:**
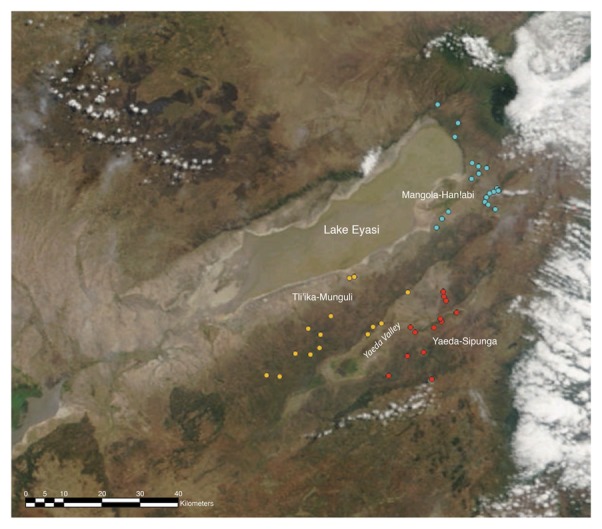
Eastern Hadza camps where interview subjects and target individuals resided in 2012, with the three regions defined by point color. In the Center is Lake Eyasi, Tanzania. Satellite image from NASA Earth Observatory.

The Hadza of Tanzania continue to forage on foot with bows, small axes, digging sticks, and carrying slings, without the aid vehicles, guns or other modern equipment. They share a common isolate click language and a set of unique customs (see File S1 for details). The Hadza were first encountered by Europeans in the late 19th century, and have been the subject of extensive anthropological research [23]. Archaeological evidence from Mumba cave attests to hunter-gatherers living in the Lake Eyasi area for at least 60,000 years [24], and genetic studies of the Hadza reveal their high genetic distance from other east African populations, their ancient shared ancestry with other African hunter-gatherers, and evidence of recent intermarriage with neighboring Bantu and Cushitic speaking neighbors [25]. The tribe now resides in an area of about 3,000 km^2^ and the Eastern Hadza sub-tribe dwells southeast of Lake Eyasi, in three distinct regional clusters, (MH, YS, TM). During the 2012 interview period, the Eastern Hadza consisted of approximately 950 individuals living in 49 named bands. GPS coordinates show these bands ranged in distance from 200 meters to ∼80 km apart, with mean distance about 35 km (Figure 2, S2 in File S1).

### Interviews

Inter-band interaction was assayed by asking Ache and Hadza subjects “yes/no” questions about social contacts with randomly chosen same-sex “target” adults (1201 same sex dyads) during specified time periods [see File S1 for more details]. Interaction rates reported in this paper represent the probability of random same sex adult dyads having interacted in the specified way at least once during a specified time period.

Between 2009–2010 KH and a native assistant conducted interviews with Northern Ache adults who had been ≥15 years old in 1970 (the year prior to first peaceful contact). Each taped interview consisted of one subject and one same-sex “target” who was chosen randomly from a list of all adults that were alive between 1965–1970 (169 men, 146 women). At the beginning of each interview we identified the “target” individual to the subject by specifying the target's unique nickname, or identifying a unique set of the target's close kin. We interviewed subjects asking 40 questions about the target's prior interaction with the subject. Seven questions on the interview are not reported in this paper because they refer only to agonistic interaction which address a different set of issues than we consider here. The final list of questions is shown in Table S1 in File S1. Each interview question could be answered as “yes” or “no”. Informants were asked about interactions only during the time period during which the interviewee was adult, and prior to first peaceful outside contact. Previous reliability studies have shown that informant recall over this time period, for group co-membership is quite accurate [17], [26], and that life history event data (births, deaths, marriages, ages, cause of death) obtained by interviews covering this time period are very reliable when reports by multiple informants are compared [22]. The interview questions asked of each subject included items like “Did target ever share meat with you?”, “Did you ever have a conversation with target?”, “Did target ever hunt or gather with you”? The final sample from the Ache consists of 351 interviews with 32 different subjects and 88 different target individuals (see Table 1).

**Table 1:** Characteristics of the Ache and Hadza study samples.

	Ache	Hadza
Population during study period	560	950
Residential bands during study period	∼20	49
Band location	mobile	tethered
Metaband rituals	yes	no
Tribal core home range	5,270 km^2^	3,000 km^2^
Same sex dyads interviewed	351	850
Different interview subjects	32	75
Different targets	88	400
Percent male dyads in sample	72%	52%
Target identified by	nickname, kin	photo
Interaction period	before 1970	2005–2012
Interaction questions	40	15

In 2012 BW and assistants administered a modified shorter version of the interaction survey to Eastern Hadza subjects. That interview contained 15 questions, 13 of which were identical to questions KH had asked the Ache (Table S1 in File S1), and these questions constituted the database for all subsequent analyses. 850 interviews were conducted with 39 men and 36 women over age 25, residing in 22 separate residential bands. Subjects were asked to report only interactions that had taken place after the widely remembered 2005 presidential election (Jakaya Kikwete) in Tanzania.

Each Hadza interview subject was shown photos of 12 same-sex target individuals. Targets included 213 different women and 187 different men residing in 42 different residential bands. The target sample was stratified to include equal numbers of individuals from all three Eastern Hadza regions (Figure S2 in File S1 shows the connection between the camps of each subject and target). To begin the interview, researchers showed facial photographs of the target individual to the interview subject. 72% of men and 60% of women were able to name the target individual after seeing a photograph, while most others knew something about the target individual but not their name. Interview questions analyzed here were identical to those asked of the Ache (eg. “Did you ever hear target X sing a song?”, “Does target X have a affinal kin relationship to you?”).

The final interview database and key are provided (Database S1, DatabaseKey S1) with the Supporting Information.

### Models and statistical analysis

Inter-band interactions take place when members of different residential groups meet and engage in social activity. Interactions often result from targeted visits, but may also occur by chance through unintended encounters between visitors and third parties. For example, individual *A* from band 1 may visit *B* from band 2 and fortuitously encounter both individual *C* residing in band 2 (not an intended target) and individual *D* from band 3 who is simultaneously visiting band 2. In this way all *As*, *Cs*, and *Ds* in the population may eventually encounter and interact with each other. For this reason, we have assumed this process can be simplified to a random encounter model. Because the elapsed time over which an interaction could take place was variable and higher for the Ache on average (mean  = 11.2 years; range  = 1–24 years) than for the Hadza (a constant 7 years) we need to estimate rates of interaction per unit time for direct comparison. To estimate yearly rates of interaction for both groups, we assumed that the interaction rate of each pair of individuals was constant through time, and then examined whether the baseline yearly rates are higher when subject and target were genetic or affinal kin or were partners in a ritual relationship. This was formalized by setting
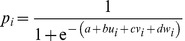
(eqn. 1)where *p_i_* is the probability of an interaction each year for pair *i*, and *u_i_*, *v_i_* and *w_i_* are dummy variables representing close genetic kinship, affinal kinship, and ritual relationship. We converted genetic coefficient of relatedness into a binary variable, close kin vs. distant or non-kin, so that the magnitude of effect of this variable could be compared directly to affine, and ritual relationship, which are also binary variables. In the statistical model *u_i_* equals one when the relatedness between pair *i* is greater than or equal to 0.125 (first cousin) and zero otherwise, *v_i_* is one when an affinal kinship term was reported for pair *i*, and zero if not, *w_i_* is one when a ritual relationship was reported for pair *i*, and zero otherwise. Positive values of the parameters *b, c* and *d* mean that genetic or affinal kinship or ritual relationship increase the rate of interaction. The parameter *a* gives the baseline yearly rate of interaction between individuals who are neither kin, affines, nor in a ritual relationship. With these assumptions, the probability that a pair of individuals has a least one interaction during *t* years, *P_i_*, is given by:
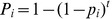
(eqn. 2)and this can be used to calculate the likelihood of the observed data from a sample of dyads.

The values of the coefficients *a*, *b, c* and *d* were estimated by finding the values that maximize this likelihood (see File S1 for computational details). Bootstrapped standard errors for these coefficients were calculated by resampling with replacement from the full dataset (1000 repetitions). The estimated yearly rates of interaction for each pair of individuals and each activity were then computed by substituting the estimated coefficients and the reported kinship and ritual relationship values for that dyad into equation 1. Mean yearly rates of interaction for each population were computed by averaging the rates for all dyads in that population. Confidence intervals for the yearly rate estimates were calculated using the joint distribution of coefficient values derived from the bootstrap analysis. The mean yearly rate of interaction was computed for each combination of coefficients with support, and the probabilities of each interaction rate were summed yielding probability distributions for the estimated mean rates of interaction for Ache and Hadza. As a guide to the significance of differences between the Ache and Hadza mean rates of interaction, we used these distributions to calculate whether the probability that the means were actually the same was less than the conventional threshold value 0.05.

## Results

### Correlates of interaction

Reported interactions were divided for convenience into five categories: 1) simple association, 2) intimate association, 3) caretaker, 4) cooperator, and 5) cultural model. Statistical analyses (below) suggest that ritual relationship in the most important predictor of increased interaction in all categories. The proportions of subjects that reported specific types of interaction with target individuals are quite high for both study populations, with probabilities of having camped together, conversed, shared meat, shared vegetable, or watched target make a tool at least once, all around 90% for Ache dyads who were adults during an average elapsed time of ∼11 years prior to peaceful contact (Table S1 in File S1, Figure S3 in File S1). Hadza dyadic interaction probabilities reported for the same activities (over the 7 year period covered by interviews) were all lower than the Ache, but still in the range of 30–40%.

Ache and Hadza interaction data were fit to eqn. 1 in order to estimate yearly interaction rates and determine if genetic or affinal kinship or ritual relationships are associated with higher interaction rates between random adult dyads in each group. These independent variables are not collinear (Table S3 in File S1). In both groups 5–10% of random dyadic pairs were “closekin” (r≥0.125), and another 15–20% were “affines” (Table 2, Table S2 in File S1). Ritual relationships were also reported in both groups. Just under 20% of Ache adults call each other by ritual terms such as “jary”, “chave”, “upiare”, “tapare”, “mondoare”, “kaviru”, “kmanove”, “mubuare” etc. All these terms are associated with birth and puberty rituals and are associated with a specific ritual role (eg. the one who cut the umbilical cord, the one who washed the newborn, the one who held the newborn, etc.). These named dyadic relationships imply rights and obligations of mutual support according to Ache social norms. Hadza dyadic named ritual partners do not exist, but people do sometimes participate together in sacred epeme dance and meat consumption rituals. Co-participants in these rituals made up about 16% of the dyads in the Hadza sample. While Ache and Hadza ritual relationships are different in character, both types of ritual relationships define and reinforce patterns of behavior between individuals, and hence may influence patterns of inter-band interaction.

**Table 2:** Values for independent variables in dyadic interaction interviews.

Indep	Question from which is derived	Percentage of sample		
Var.		All	Ache	Hazda
Closekin	Ego and target closely related (genetic coefficient r≥0.125)	5.86	9.4	4.47
Affine	Ego employs affinal term for target (ie. spouse's sibs or parents)	16.31	19.94	14.88
Ritual	Ego and target have ritual relationship	17.07	20.23	15.76
Elapsed	Mean years at risk of adult interaction with target	8.17	11.16	7
	(during pre-contact forest period for Ache)			
Sex	Female to Female dyadic relationship	42.41	27.92	48.1
	Male to Male dyadic relationship	57.59	72.08	51.9
Ethnicity	Ache	28.19	(100)	(0)
	Hazda	71.81	(0)	(100)

Maximum Likelihood estimates of the coefficients b, c, and d, measure the effect of each of the independent variables with the other two variables controlled. These are presented in Table 3 for the 13 interaction types recorded for both groups (detailed analyses of alternative models are provided in Tables S4–S13 in File S1). The most important predictor of dyadic interaction in the Ache interviews was simply the elapsed time that subject and target were adults in the precontact period (the model in Tables S4–S8 in File S1 that contains only the constant term). Genetic kinship was not associated with significantly higher interaction rates for the Ache, but affinal kin did show significantly higher interaction rates for two questions. Ache dyads with ritual relationships interact significantly more often for 5 of the 13 questions analyzed. Among the Hadza elapsed time was constant and genetic kinship was significantly associated with higher interaction rates for 12 of 13 questions. Affinal kin experienced higher interaction rates for 11 of 13 questions, while Hadza dyads with common participation in the epeme ritual showed higher interaction rates for all 13 questions.

**Table 3:** Maximum Liklihood Estimates of the beta coefficients for genetic kin (closekin), affinal kin, and ritual relations from equation 1.

Group	Dep Var	Question	Ache			Hazda		
			closekin	affine	ritual	closekin	affine	ritual
**Associate**	q6	Have you spoken with target?	0.525	0.031	0.413	1.490*	1.308*	2.862*
			(0.942)	(0.268)	(0.433)	(0.323)	(0.169)	(0.695)
	q8	Did target sleep in your camp?	0.048	−0.003	0.358	1.133*	0.834*	2.193*
			(0.971)	(0.351)	(0.469)	(0.348)	(0.218)	(0.176)
	q10	Have you joked with target?	−0.313	0.409*	−0.114	0.637	1.444*	1.671*
			(0.288)	(0.205)	(0.204)	(0.376)	(0.213)	(0.219)
**Intimate Associate**	q22	Has target given you a non food gift?	−0.131	−0.340	0.478*	1.098*	0.534*	2.098*
			(0.342)	(0.268)	(0.232)	(0.364)	(0.212)	(0.165)
	q34	Did target ever groom you?	−0.298	−0.287	0.772*	1.053*	0.079	2.245*
			(0.820)	(0.340)	(0.273)	(0.367)	(0.287)	(0.220)
**Caretaker**	q39	Did target give you food when you were sick or injured?	0.406	0.264*	0.705	1.015*	0.368	1.988*
			(0.388)	(0.297)	(0.270)	(0.348)	(0.227)	(0.190)
**Cooperator**	q18	Has target shared non meat foods with you?	−0.074	−0.026	0.888	0.973*	0.566*	2.087*
			(0.385)	(0.302)	(0.457)	(0.374)	(0.208)	(0.163)
	q19	Has target shared meat with you?	−0.071	−0.210	0.962*	1.262*	0.709*	2.133*
			(0.377)	(0.259)	(0.474)	(0.339)	(0.216)	(0.167)
	q23	Did target ever lend you something?	−0.178	−0.528*	0.490*	1.122*	0.650*	1.994*
			(0.285)	(0.231)	(0.202)	(0.297)	(0.201)	(0.158)
	q24	Did you ever hunt/collect roots with target?	−0.216	−0.103	0.188	0.843*	0.696*	2.189*
			(0.281)	(0.202)	(0.190)	(0.384)	(0.196)	(0.168)
**Cultural Model**	q9	Have you heard target sing?	−0.414	−0.277	0.272	0.965*	0.850*	1.988*
			(0.294)	(0.219)	(0.213)	(0.361)	(0.192)	(0.164)
	q17	Has target shared news with you?	0.174	−0.186	0.416*	1.565*	0.935*	2.299*
			(0.297)	(0.236)	(0.207)	(0.318)	(0.218)	(0.207)
	q31.1	Did you ever watch target make a tool?	0.045	−0.146	1.229	1.075*	1.176*	2.358*
			(0.967)	(0.349)	(0.713)	(0.413)	(0.197)	(0.209)

Note  =  Bootstrapped standard errors in parenthesis (over 1000 repetitions).

* =  estimate significantly different from zero at the 5% level.

**Figure 3:**
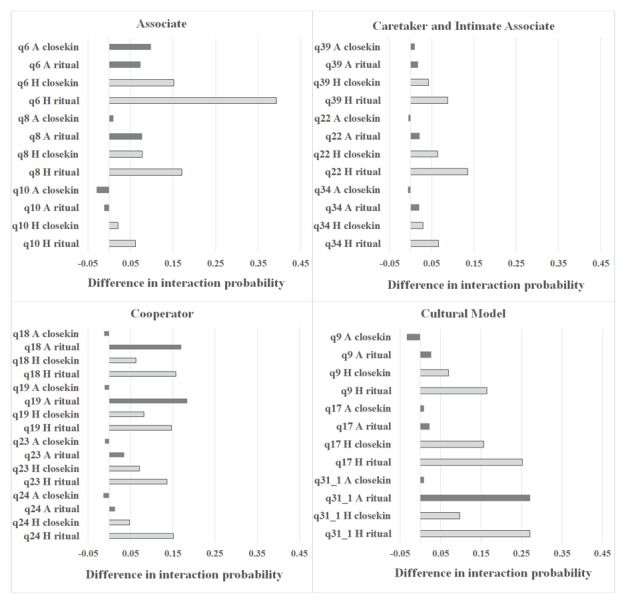
Comparison of the effect of “closekin” and “ritual relationship” on the predicted probability of engaging in specified interaction types. Each bar represents a the average difference in the probability of interaction calculated as: (P(y)|closekin  = 1) – (P(y)|closekin  = 0) for “closekin”, and (P(y)|ritual  = 1) – (P(y)|ritual  = 0) for “ritual relationship”. Probabilities are calculated by averaging values over all dyads while keeping closekin or ritual fixed at 1 or 0, and *t* = 1. Dark bars represent Ache, light bars represent Hadza.

When we compare the overall effects of kinship and ritual, it is clear that ritual relationship is far more associated with interaction than is kinship for both study populations. The effect size (beta coefficient) is greater for ritual than kin for 11 of the 13 questions for the Ache, and 13 of 13 questions for the Hadza. Because most Ache ritual relationships were intiated at birth or puberty but interaction took place in adulthood, we can assume that the ritual relationship promoted interaction (not vice versa). Among the Hadza, individuals must spend at least some time in the same camp in order to jointly participate in the epeme ritual, hence common coresidence might produce an association between ritual co-participation and interaction frequency in other realms. However, co-residing in the same camp is not always a significant predictor of interaction for the Hadza, and even when coresidence is controlled by multiple regression, ritual relationship is still an important predictor of interaction (Tables S9–S13 in File S1). In most cases for either Ache or Hadza the effect of ritual on interaction rates is more than twice as great as that of either type of kinship (eg. Figure 3).

### Yearly Interaction Rates

Yearly interaction rates for each of the sample dyads were calculated by solving for *p_i_* in equation 2 with the effects of the three independent variables determined by the maximum likelihood estimates for eqn. 1 as reported in Table 3. These yearly interaction rate estimates were then averaged for all dyads to obtain a population mean yearly interaction rate for each type of interaction covered by the interviews.

Ache yearly probability of interaction for common activity types (conversing, sleeping in same camp, sharing food, watching tool manufacture) ranged from about 0.20 to 0.30 per anum, and were generally significantly higher (2–5 fold) than Hadza rates (Table 4, Figure S4 in File S1). Medium frequency interactions (joking, listening to the target sing) showed probabilities of 0.10 to 0.12 per year among Ache, about 2 to 4 times higher than the Hadza. Finally, rare interaction types (hunting/collecting together, giving non-food gifts, grooming, lending a tool, sharing important news, and caring for sick/injured individuals) showed generally low annual probabilities for both the Ache and the Hadza (0.02–0.08 per dyad per year), with each group showing some higher rates for specific interactions (Table 4). In sum, 9 of 13 Ache yearly rates were significantly higher than for the Hadza and 2 of 13 of the Hadza rates (transmitting news, feeding when incapacitated) were significantly higher than for the Ache.

**Table 4:** Proportion of dyads reporting interaction of specified type, and the yearly interaction rate estimated from equation 2, with effects of kinship and ritual relationship controlled.

Dep Var.	Question to Interviewee	Proportion of sample interacting^#^		Yearly interaction probability^##^	
		Ache	Hadza	Ache	Hadza
	**Associate**				
q6	Have you spoken with target?	0.87	0.40	0.215*	0.119
q8	Did target sleep in your camp?	0.93	0.28	0.293*	0.058
q10	Have you joked with target?	0.71	0.17	0.117*	0.028
	**Intimate Associate**				
q22	Has target given you a non food gift?	0.37	0.24	0.040	0.047
q34	Did target ever groom you?	0.22	0.13	0.022	0.020
	**Caretaker**				
q39	Did target give you food when you were sick or injured?	0.21	0.19	0.021*	0.031
	**Cooperator**				
q18	Has target shared non meat foods with you?	0.88	0.28	0.219*	0.055
q19	Has target shared meat with you?	0.87	0.26	0.217*	0.051
q23	Did target ever lend you something?	0.54	0.26	0.075*	0.050
q24	Did you ever hunt/collect roots with target?	0.78	0.26	0.068*	0.051
	**Cultural Model**				
q9	Have you heard target sing?	0.69	0.31	0.102*	0.063
q17	Has target shared news with you?	0.44	0.36	0.051*	0.089
q31.1	Did you ever watch target make a tool?	0.90	0.38	0.277*	0.093

Note: #  =  raw proportion of sample interacting for each interaction type. ##  =  mean yearly probability of interaction based on eqn 1 & 2 with variables ritual, closekin and affine, plus a constant term and averaged over all dyads. *  =  Hazda and Ache mean rates are significantly different at the 5% level (see File S1 for details on calculations).

### Lifetime Interaction Partners

Our model assumes that interactions take place at a constant rate, hence the cumulative probability of at least one interaction over time is a negatively accelerated monotonically increasing function. For many of the interaction types that we examined the probability of at least one interaction between random same sex adult dyads becomes quite substantial after ten years or more (Figures S5, S6 in File S1). Combining estimated yearly interaction rates with the population size and life tables (Table S15 in File S1) allows us to estimate the expected number of lifetime adult interaction partners for Ache and Hadza adults.

Assuming a stable age-structured population the mean number of lifetime adult interactants (*N*) can be expressed as:
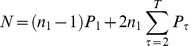
(eqn. 3)


**Figure 4:**
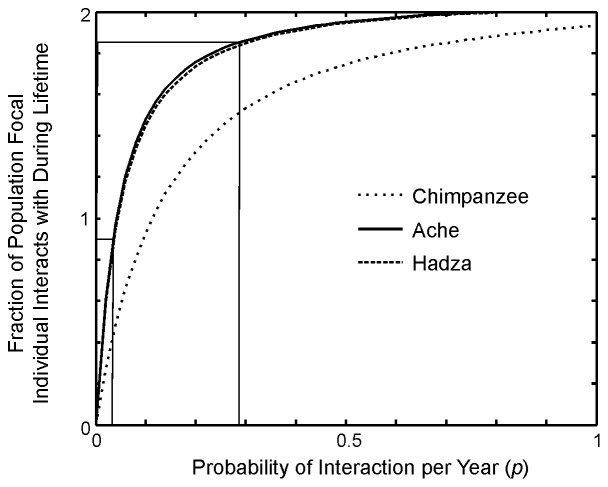
Fraction of the adult male standing population that interact at least once over a lifetime plotted by different yearly rates of interaction (p) covering the range of yearly interaction rates listed in Table 4, based on the adult survival table for Ache, Hadza, and Chimpanzees. The shaded area shows the range of yearly interaction rates of the hunter-gatherer populations.

Where *n_1_* is the number of adults in the first yearly adult age cohort of the interacting population, *T* is the maximum age to which an adult survives, and *P_τ_*, the yearly probability of an interaction with a target individual that is *τ* years older than the subject, is a function of both adult survival rates and yearly interaction rates (see File S1 for derivation). Since the number of individuals who enter the first age cohort is proportional to the total size of a stable population, the number of expected lifetime interactants is approximately a linear function of size of the potential interaction population. With moderate interaction rates and low mortality, the number of lifetime interactants approaches 2*n* since there are *n* older interactants when ego reaches adulthood and ego will meet about *n* younger new interactants before dying (Figure 4).

Because recent theoretical work suggests that interaction network size can determine whether cultural traits accumulate complexity [11], [12] we are particularly interested to compare the social universe of hunter-gatherers to chimpanzees. Our calculations suggest that the lifetime number of interactants for male Ache and Hadza hunter-gatherers is more than an order of magnitude higher than that expected for male chimpanzees (female dispersal from the natal community makes this calculation difficult for chimpanzee females). Precontact Northern Ache men, for example, are expected to have hunted with 206 men, conversed with 290 men, and watch tool making by 302 different men in a lifetime (Table S16 in File S1). Eastern Hadza men are expected to converse with 427 other Hadza men, hunt with 303 men, and watch tool making by 395 different men in a lifetime. In contrast, chimpanzees living in typical communities of 11 adult males are expected to interact with a mean of only 21 other adult males in a lifetime (Table S16 in File S1).

The relationship between yearly probability of interaction and expected proportion of the population that will interact in a lifetime asymptotes quickly, and even low rates of interpersonal interaction among hunter-gatherers lead to lifetime interactions with most other adults (Figure 4). This is because the human adult lifespan is so much longer than that of chimpanzees. The average expected time that both members of an Ache or Hadza dyad who enter adulthood together will both still be alive is about 27 years while the average expected time that two male chimpanzees from the same community, entering adulthood together, are still both alive is only 6 years.

## Discussion

Among the Ache and Hadza, frequent visiting and long lifespans mean that adults typically interact with more than three hundred same-sex adults during their lifetimes. This implies a social universe of about a thousand individuals, when opposite-sex adults and children are included. Recent work on the San Bushmen also suggests a similarly high number of significant interactants [27]. Additionally, close companions often interact with a somewhat different set of individuals, so that the total number of indirect interactants that each individual hears about repeatedly in detailed stories, and could expect to possibly meet some time during their lifetime is clearly more than 1,000. This is a much higher number of individually known social interactants than reported for any other primate, and possibly more than any other species on earth. It is also much greater than the predicted 150 significant social interactants (known as “Dunbar's number”) that was extrapolated from primate brain by social group size regressions [6], [7]. It should not surprise us that humans have more relationships than their brain size alone predicts, as humans alone use language and symbolic devices to store information about potential relationships. The main reason why human's interact with so many more individuals than other apes is because: 1) human lifespans are much longer, and 2) interaction between neighboring and distant residential social units is extensive.

An important question is when and how the meta-band social structure arose in hominin evolution. We agree with other authors that pair-bonding, paternal investment, and habitual marital exchange between kin groups [16], [28] are probably the key to this shift. Our results suggest that affinal kinship is about as important as genetic kinship for interactions between hunter-gatherer adults. Likewise, the fact that ritual relationship is a strong predictor of inter-band interaction in the Ache and Hadza is congruent with the idea that cultural institutions often expand interaction networks in hunter-gatherer societies [18],[29]. For example, the fact that the Ache organize multi-band club fight rituals each year whereas the Hadza have no multi-band rituals is an important reason why Ache inter-band yearly interaction rates are higher.

We are not yet certain to what extent the Ache and Hadza are representative of other hunter-gatherers. First, both study populations are small and encapsulated, with hostile relations with neighbors. In other regions of the world, relations with surrounding ethnic groups may be more peaceful allowing even larger interaction networks. Additionally, the Hadza and Ache practiced no long distance trade and the spheres of interaction with outsiders may be narrower than among other world foragers where trade networks were important. On the other hand, the Ache and Hadza exhibit no land ownership or territoriality. In other foraging societies land ownership was important and structured inter-group interaction in ways that might restrict interaction networks.

We should also note that the Ache and Hadza differ between themselves. Ache maintained higher yearly interaction rates, yet the Hadza live in a larger ethnolinguistic unit with more total interactions possible over long time periods. The higher Ache yearly interaction rates appear to be due to both ritual activities (mentioned above) and different spatial social structure. Hadza bands are geographically localized but Ache bands ranged freely throughout the entire tribal territory. Hadza, but not Ache foragers also show a more consistent sex difference with Hadza women generally showing lower interaction rates than Hadza men (Table S14 in File S1, Tables S9–S13 in File S1).

**Figure 5:**
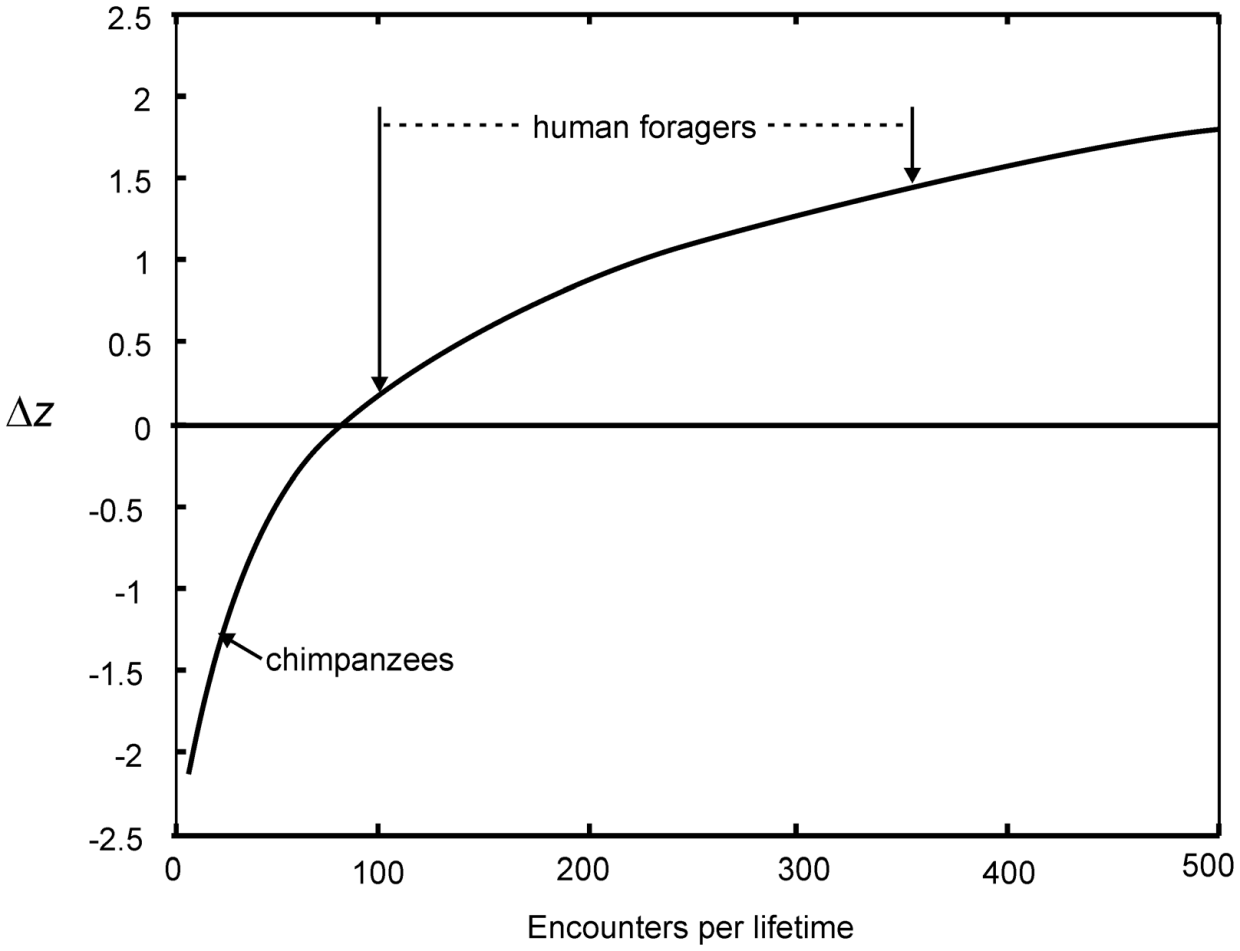
Mean change across one generation of social learning, in the cultural adaptiveness of a trait of moderate complexity following Henrich (*11*: Fig. 3). The α/β ratio of the model is 5, indicating a moderate mean decrease in cultural adaptedness through initial copy error followed by moderate dispersion of cultural adaptedness (z values) of copiers due to random error and guided innovation. With success-biased imitation, human foragers, with their large number of observed models (range from Table S16 File S1 is indicated), accumulate improvements each generation (Δz>0), whereas chimpanzees, with a smaller number of lifetime interactants are not able to maintain or improve the initial cultural traits (Δz<0).

The difference in lifetime interaction partners between humans and other apes has important implications. First, hominin brain expansion may be primarily explained by the memory requirements of storing critical details about an increasingly large number of social partners [6]. Second, mathematical models of cultural evolution suggest that a tenfold increase in the number of potential cultural models, can allow populations with skills and technologies that are difficult to copy to achieve a ratcheting up of cultural adaptation while populations with fewer individuals to imitate would never succeed in doing so [11]. For example, our simulation shows that for a cultural trait of medium difficulty (moderate imitation error and moderate innovation), hunter-gatherers that prefer to copy the most competent individual they observe, could easily maintain and improve the practice or technology whereas chimpanzees, would not be able to maintain the trait nor improve on it through time (Text in File S1, Figure 5). Simply put, chimpanzees are unlikely to observe a cultural trait much better than that displayed by the previous generation. Humans, by observing more individuals in a lifetime, are more likely to ecounter one person who has innovated a significantly better cultural trait than was present in the previous generation. By imitation of the best trait, progressive improvement is possible. Thus, the reason why humans, but not chimpanzees have cumulative culture may be partially due to social structure as well as cognitive differences.

Third, many of the inter-band interactions we recorded were cooperative in nature. This should lead researchers to focus on mechanisms that can explain cooperation in groups of hundreds of unrelated individuals. Finally, the large interaction networks that characterize ancestral hunter-gatherers have important implications for infectious disease transmission. How this affected the evolution of the human immune system and health practices in our ancestors remains to be investigated.

## Supporting Information

File S1 **Includes Text S1; Figures S1–S6; Tables S1–S16; References S1.**
(DOC)

Database S1 **Database for Analyses.**
(XLS)

DatabaseKey S1 **Key for Database.**
(DOCX)
