# Supplementary material for: Hunter-Gatherer Inter-Band Interaction Rates: Implications for Cumulative Culture
Source: PLoS One. 2014 Jul 21;9(7):e102806. doi: 10.1371/journal.pone.0102806 (PMC4105570; doi:10.1371/journal.pone.0102806)
Supplement: File S1 — Includes Text S1; Figures S1–S6; Tables S1–S16; References S1. (DOC) [file pone.0102806.s001.doc]

**Inter-band Interaction Rates: Electronic Supplementary Material**

**Authors:** Kim R. Hill^1^*, Brian Wood^2^, Jacopo Baggio^1^, A. Magdalena Hurtado^1^, Robert Boyd^1^

**Supplementary Information**

**Ethnographic Background**

The Ache are a population of hunter-gatherers that roamed the forests of eastern Paraguay until pacification in the 1970s. They are best known scientifically for studies on foraging choices, sexual division of labor, food sharing, life history evolution, and cooperation [1-6]. The Ache “tribe” shares a single mutually intelligible language of the Tupi-Guarani language family and all dialectal sub-tribes of Ache show cultural features missing from the surrounding agricultural tribes of the region. The Ache had no pan-tribal political or religious leaders and never gathered together in one place for any political or religious functions. There is neither recollection nor historical account of any peaceful interaction between Ache and surrounding Guarani agricultural tribes going back to the 17^th^ century, despite the fact that they belong to the same macro language family. There was no trade across Ache-Guarani tribal boundaries, and the small amount of gene flow that might have occurred was due to violent encounter (warfare, kidnapping, rape). Genetic studies instead suggest the Ache might have originated as a more ancient hybrid population with horticulturalist Guarani male contribution and female contribution from the Ge speaking Kaingang hunter-gatherers [6].

Within the Ache tribe there were four regional “sub-tribes” in the 20^th^ century (Figure S1), defined by dialect and minor cultural differences (Northern, Yvytyruzu, Ypeti, and Ñacunday Ache respectively). The Northern and Yvytyruzu Ache split from each other in the 1940s when the modern road to Ciudad del Este was built. The more southerly Ypeti and Ñacunday Ache have an ambiguous historical relationship to each other and to the Northern groups. Informants report that there were no peaceful interactions between any of the four Ache sub-tribes by the mid 20^th^ century, but sub-tribes must derive from a fairly recent common ancestor (probably from the early 19^th^ century) because all speak mutually intelligible dialects of the Ache language.

Ache sub-tribes were each subdivided further into multiple residential bands that camped together and formed the basis of daily living. Bands were highly mobile, never remaining in one location for more than a week. Because of visiting and migration, band composition was stable over periods of months but not years. The Northern Ache, subjects of the present study, constituted the largest Ache sub-tribe, consisting of about 560 people living in 18-20 residential bands during the last decade before peaceful outside contact (1960-1970). Northern Ache bands were usually dispersed throughout their core home range of ~ 5,270 km^2^ (Figure 1, main text), but during some years a few of the bands might be far to the southwest in the larger sub-tribe maximal range. Informants report that the different residential bands were generally located at distances of 10-30 km from each other, but sometimes might roam as far as 150 km apart when bands made temporary use of distant areas of their ~14,630 km^2^ maximal sub-tribal range.

The Hadza foragers of Tanzania were first encountered by Europeans in the late 19th century, and have been the subject of extensive anthropological research [7] Archaeological evidence from Mumba cave attests to hunter-gatherers living in the Lake Eyasi area for at least 60,000 years [8]. Genetic studies of the Hadza reveal their high genetic distance from other east African populations, their ancient shared ancestry with other African hunter-gatherers, and evidence of recent intermarriage with neighboring Bantu and Cushitic speaking neighbors, the Sukuma, Isanzu, and Iraqwa peoples [9]. The earliest evidence of pastoralism in the area dates to 2,500 years BP and agriculture only 600 years BP [10]. The Hadza slowly increased their interaction with neighboring tribes and European colonists and anthropologists during the 20th century. Here we define Hadza as those who speak Hadzane, a language isolate sometimes categorized as Khoisan due to its use of click consonants [11]. Today about 950 Hadza live to the southeast of Lake Eyasi, and a smaller number (150-250) live to the to west of the lake. The two regions have slight cultural and linguistic differences. Our fieldwork in 2012 was carried out among the Eastern Hadza sub-tribe, who in early 2012 lived in 49 named residential groups spread across an area of approx. 3,000 km^2^ (Figure 2, main text).

Though the Eastern Hadza have no territorially defined political groups, they do refer to other Hadza as living among a set of geographic areas, which we use to define three distinct regions: Mangola-Han!abi (MH), Yaeda-Sipuga (YS), and Tli’ika-Munguli (TM). Between January and May of 2012, BW visited every Eastern Hadza residential group and enumerated 323, 368, and 237 living Hadza in the three regions, respectively (we assume a 2% undercount in the population census, owing to the Hadza’s mobility).

Within the Eastern Hadza population, some residential groups practice nearly full-time foraging (approximately 250 individuals, most in the Tli’ika-Munguli region) while others subsist opportunistically from foraging, very small-scale farming, and ethnotourism. A few Hadza in the Yaeda-Sipunga area work seasonally protecting maize fields. Those Hadza who farm maintain very small (1/4 hectare or less) maize fields, with frequent crop failure and small harvests the norm, requiring these groups to continually forage for wild foods. Groups engaged in ethnotourism also continue to derive a significant portion of their diet from foraging. The Hadza forage on foot with bows, small axes, digging sticks, and carrying slings, without the aid vehicles, guns or other modern equipment. Among those Hadza that practice traditional foraging methods full-time, over 95% of their calories come from wild foods, including tubers, berries, small- and large-game, baobab fruit, and honey [7].

Methods

1. Interaction interviews

Between 2008-2010 KH and a native assistant conducted interviews with a sample of Northern Ache adults who had been ≥15 years old in 1970, the year of first peaceful outside contact. Each taped interview consisted of one subject and a “target” individual who was chosen randomly from a list of all Northern Ache that were both alive and adult between 1965-1970 (169 men, 146 women). We initially asked 48 questions, about the target’s prior interaction with the subject and several more about the dyad’s kinship and ritual relations. Eight of these were eliminated early in the interview process because they were deemed redundant or ambiguous. Each remaining interview question; “did specified interaction take place?” could be answered as “yes” or “no”, and we reminded interview subjects that the questions being asked referred only to the time period when the interviewee was adult, and before the first peaceful contact that took place in 1970. Raw proportions of “yes” answers to 33 of these questions (7 questions about agonistic interactions have been eliminated from this table) are reported in Table S1. The final sample from the Ache consists of 351 same sex interviews (559 total interviews including cross-sex interviews not analyzed in this paper) with 32 different subjects and 88 different target individuals. No Ache subject ever reported knowing about the existence of any adult in the population that was not known to them by name and nickname.

The use of interview data about interactions in the precontact period for the Ache is of some concern. There were no randomly chosen dyads among the Ache for whom roles of subject and target were reversed. This is because the majority of targets among the Ache were deceased individuals. We would have preferred to interview a stratified random sample that included some living dyads, in reverse order for verification, but it was hard to locate individuals living in six different communities when many are on forest treks during days that we visited. We had intended to follow up with reverse interviews near the end of this study but were unable to return to the field after 2012 due to dangerous conditions. We have previously assessed interview reliability three times. In our Ache Life History book^21^ we report that independently interviewed adults showed good agreement on numbers of children produced by married pairs in the pre-contact period, and on causes of death for a large number of deceased individuals (Hill and Hurtado 1996 chapter 2). Second, interviews of band membership in the precontact period show good agreement between informants on who was living together in their band at the time that some specified event took place^17^. Finally, interviews at the reservation about what game had been killed on trek in previous weeks agreed well with the actual observed game harvest on those treks^25^. We see no reason to expect biased inaccuracies in informant recall about these interaction events. The interactions are neither controversial nor secret, and are openly witnessed. Informants clearly distinguish the frequency of some interactions vs others (eg conversing together is frequent, bathing together is infrequent). Perhaps we might expect a general forgetfulness about events in the past but that would simply lower the annual interaction rates. If so, our results are a conservative estimate, still suggesting that humans interact with many more individuals than do chimpanzees (or other primates). We see no particular reason to believe that interactions with ritual partners would be selectively over-reported relative to interactions with close kin or affines, so there is no reason a priori to expect the pattern that we report of ritual relations interacting more frequently than kin. Ultimately, however we hope that this study will motivate other similar studies which may provide better verification of interview based data in order to determine whether the patterns we report here are robust.

In 2012 BW and assistants administered a similar but shorter version of the Ache questionnaire to Eastern Hadza subjects. This interview included 13 questions that were worded identically to ones on the Ache interview and were employed for all subsequent analyses (Table S1). For these interviews Hadza subjects and targets were always the same sex. Interview subjects were asked to report only interactions that had taken place after the widely remembered 2005 presidential election (Jakaya Kikwete) in Tanzania. Eastern Hadza interview subjects (n = 39 male, 36 female) resided in 22 separate residential bands, including 27, 26, and 22 subjects residing in the Mangola-Han!abi (MH), Yaeda-Sipuga (YS), and Tli’ika-Munguli (TM) regions respectively. Through fieldwork BW has accumulated photographs of 400 Eastern Hadza adults aged 25 and older, and these 400 individuals (213 women, 187 men) comprised the pool of possible targets for the interaction interviews.

Each Hadza interview subject was shown 12 target individuals. Target individuals in the Hadza sample resided in 42 different residential bands and the sample was stratified to include equal numbers of individuals from all three Eastern Hadza regions. In Figure S2, we show the connection between the same camps of each interview subject and target. To begin the interview, researchers showed facial photographs of the target individual to the interview subject. 72% of men and 60% of women were able to name the target individual after seeing a photograph, while a most others knew something about the target individual but not their name.

**Database**:

We provide the full database used in analyses, along with a key for the variable columns as a link at the end of the supplemental information.

2. Statistical modeling of interaction rate and determinants of variation

While Hadza interviews specified the time period of interaction as the seven years from 2005-2012, the Northern Ache interactions took place between individuals who were “at risk” of interacting over varying elapsed time periods in the 1950s and 60s. Because interviewees and targets might be different ages, and some died prior to the contact event, the period of “risk” for an interaction between the dyads began the year that both target and interviewee were 15 years old, and ended at contact, or when the target individual died (which ever came first).

Because the elapsed time over which an interaction could take place was variable and higher for the Ache (mean = 11.16 years; range = 1 years to 24 years) than for the Hadza (a constant 7 years) direct comparison of tabulated interview interaction probabilities for the two groups is not informative. In order to compare interaction rates for each activity type we need to estimate the yearly interaction probabilities for each type of interaction and for each ethnic group. To do this we assumed a constant probability of dyads interacting each year (random encounter model) and then examined a variety of independent variables to determine how they affect this probability. Our main theoretical interest is whether genetic kin, affines, or individuals with ritual relationships interact more often than dyads with no such relationships, but we also examined two other independent variables: the effects of living in the same camp during a reported census period and sex of the interview dyad. The population average values for the independent variables are shown in Table S2. The correlations between independent variables, shown in Table S3, indicates that independent variables are not collinear and can be examined together in a single multivariate model.

With a constant rate of interaction, the cumulative probability of any specific dyad *i*, engaging in interaction type *j* at least once over the elapsed period of exposure (*t*) is calculated as:

$P_{t,j,i}=1-(1-p_{jid})^{t_{i}}$ (eqn S1)

where *p_t,i,j_* is the yearly probability of interaction type *j* at time *t* for dyad *i*, *t_i_* is years covered by the interview with both members of the dyad *i* alive and adult and

$p_{jid}=\frac{1}{1+e^{-(b_{j0}+\sum_{d} b_{jd}x_{id})}}$ (eqn S2)

where the *x_id_* = independent variables that may affect interaction rate and the *b_jd_* are coefficients that control the effect of that variable on the rate of interaction of type *j*. The average probability of interaction of type *j* for all dyads over a specific time of exposure t is then:

$P_{t,j}=\sum_{i=1}^{N} \frac{P_{t,i,j}}{N}$ (eqn S3)

where *N* is the total number of dyads.

The values of *b_jd_* were estimated via maximum likelihood estimation with bootstrapped standard errors in Stata 9.1. This procedure selects model parameters that maximize the likelihood function. Bootstrapped standard errors are calculated in Stata 9.1 with 1000 repetitions and sampling from the original data with replacement.

In order to examine if the three theoretically proposed factors (genetic kinship, affinal kinship, ritual relationship) are associated with higher rates of interaction between dyads we collected information about dyads that included these variables and the period at risk of interaction for each dyad. Tabulation of the independent variables showed that about a third of the interview subjects in both groups report using a consanguineal kin term for the target individual but only 5-10% of the dyadic pairs are “close kin” (*r* ≥ 0.125) and another 15-20% are recognized as affines (in-laws) (Table S2). In order to directly compare the magnitude of the effect of kinship to the effect of having a ritual relationship, kinship was converted to a dichotomous variable that was assigned the value 1 if *r* ≥ 0.125, and 0 otherwise. Similarly, affinal relationships were represented by a dummy variable that took on value 1 if the pair were reported as affines. The mean genetic coefficient of relatedness for all same sex dyads interviewed (both Ache and Hadza) was between 0.02 to 0.05, similar to the mean genetic coefficient that we previously reported for coresident adults in a large sample of precontact Ache bands [12]. Hence the vast majority of dyads examined by interview were not “close kin”.

Ritual relationships were commonly reported in both groups. Just under 20% of Ache adults call each other by ritual terms such as “jary”, “chave”, “upiare”, “tapare”, “mondoare”, “kaviru”, “kmanove”, “mubuare” etc. All these terms are associated with birth and puberty rituales where adult sponsors become the “godparents” to children that they assist, and also take on a named ritual relationship with the parents of their “godchild”. Each of these named relationships is associated with a specific ritual role (eg. the one who cut the umbilical cord, the one who washed the newborn, the one who held the newborn, etc.), and all named relationships imply rights and obligations of mutual support according to Ache social norms. Ritual relationships were often the stated justification for visiting distant bands or for residential transfers. Hadza dyadic named ritual partners do not exist, but people do sometimes participate together in sacred epeme dance and meat consumption rituals. In both cases ritual relationship was represented as a dummy that took on value 1 if a relationship existed between members of the pair. Co-participants in these rituals made up about 20% of the dyads in the Hadza sample (Table S2).

Two other independent variables were considered for alternative models of the yearly interaction rate. These were observed coresidence in a prior census (samecamp), and sex of the dyad. Observed coresidence in the same camp on at least one day was likely to be associated with some types of interactions. About 4% of all Hadza dyads were residing in the same camp on the day of the interview. For the Ache dataset coresidence was evaluated over the entire dydadic exposure period, from random census data on 58 precontact bands. That census data showed that just under 20% of all Ache dyads in our interview dataset were found residing together in at least one camp on at least one day during the precontact period (Table S2).

The Maximum likelihood estimates of a null model with no predictor variables, the primary model with 3 independent variables, (close genetic kin, affinal kin, and ritual relationship) and the alternative models that include camp coresidence and sex as predictors are provided in tables S4-S13.

Examination of the Akaike information criteria (AIC) values shows that the 3 variable model is slightly better than the null model (no variables) and almost as good as the 5 variable model (but less complicated), when averaged across all questions. The AIC provides guidance on model selection, however, given the number of observations and of events/non-events for the different interaction type, overfitting is a concern. In order to avoid overfitting we take into account the minimum number between events/non-events (i.e. min [*p*, 1*-p*], where *p* is the proportion of events). Simulation studies have demonstrated that a model can lead to reasonable estimates when the sample size (in our case min [*p*, 1*-p*] of the dependent variable) allows at least for 10 observations per predictor [13]. This suggests that only 3 independent variables should be examined for Ache questions that had high frequencies of yes or no answers. Due to the limited model improvement with more than three predictor variables, and the fact that yearly interaction rates were essentially the same with both models, we decided to concentrate on the analysis of close kinship, affinal kinship, and ritual relationship.

Yearly encounter rates can be estimated using equation S1 and the three variable model. First we derive the beta coefficients for genetic kin, affinal kin, and ritual relationships from maximum likelihood estimation. Then we apply the independent variable values for each dyad in the sample to eqn. S2 and solve for *P_tj_* in eqn. S1 when *t*=1. This value for each dyad is then averaged over the entire sample to get the mean yearly interaction rate (eqn. S3) for each interaction class for Ache and Hadza adults (see table 3, main text).

3. Ethnic differences in interaction rates

The complete list of interaction interview questions and proportion “yes” responses for male-male and female-female dyads is shown in Table S1. Note that only a subset of the interview questions were asked in Hadza interviews (na = not asked). Interviews from both sexes are combined for analyses. The probability of a “yes” answer to whether a specified interaction type had taken place between dyads (*P_t,j_*) was uniformly higher among the Ache for every question asked of both groups (Figure S5).

After controlling for the effects of the three independent variables and estimating the yearly interaction probabilities analyses shows that the Ache yearly interaction rates (*p_ji_*) for different activities range from 2% to 29% per anum depending on interaction type and are higher than the Hadza yearly rates for 11 of 13 questions (text Table 3). We then developed a procedure for evaluating the likely statistical significance of those differences. Interval estimates for the yearly rates of interaction for the Hadza and Ache were calculated based on the distribution for the estimators of the coefficients. The Stata bootstrap routine produces a vector of means and a covariance matrix for the four estimators of the coefficients, the constant and the coefficients on the three dummies representing kinship, affinal relationship and ritual relationship. For each population, we created 15,000 combinations of coefficient values with substantial support. Then for each combination of coefficient values, we computed the average interaction rate over all pairs. Then assuming a multivariate normal distribution with the given covariance matrix, we summed the probabilities of all combinations of coefficients that produced the same interaction rate. This procedure yields a probability distribution for estimates of the yearly interaction rate for each population. We then used these probability distributions to calculate the probability distribution of the difference between the mean rates of interaction Ache and Hadza. We judged the observed difference to be significant if the probability that the two rates were the same was less than 0.05 (a two-tailed test).

Results of these analyses show that the rate of interaction for Hadza is significantly greater than that for the Ache on q17 (sharing news) and q39 (feeding when incapacitated). There is no significant difference for q22, q34, and for the other nine questions the rate of interaction for the Ache is significantly greater than that for the Hadza (Figure S4; text Table 3). In general we might expect Ache dyadic interaction rates to be somewhat higher because their total population is smaller. The higher Hadza rates for q17 may be due to translation problems since there is no good equivalent in the Ache language for “news” or anything close (“gossip”, “information”, etc.). Likewise, many of the Ache answered q39 by stating that they had never been sick or incapacitated in the forest period and therefore there was no chance that the target individual could have fed them while sick.

4. Expected lifetime interaction partners.

Given the measured yearly interaction rate for all dyads in a population, along with the population size, and the survival life table of adults, it is possible to calculate the expected number of lifetime interaction partners for different activity types. Here, an interaction partner is each individual with whom ego has interacted with at least once during his/her lifetime. Assume an age structured population. The time periods are labeled $t=1,2,...,T$. At each time period $n_{1}$ individuals enter at age 1. During each time period they have a probability *p* of meeting each other individual in the population. They have probability $s_{x}$ of surviving to the next period when they will be one period older. So mortality always occurs at the end the period. The probability of surviving to age $x>1$, is

$$l_{x}=\prod_{i=1}^{x-1} s_{x}$$

where by convention $l_{1}=1$ . Notice that his means that $s_{x}=\frac{l_{x+1}}{l_{x}}$ where $l_{T+1}=0$ .

Now consider a focal individual who enters the population at $t=1$ and a second “target” individual who entered the population *τ* periods previous, and thus if still alive has age *τ* at the time the focal enters the population. We want to calculate the probability that the focal interacts with the target at least once, $P_{\tau}$. To do this we calculate the probability that they never interact $Q_{\tau}$.

Start at period 1. There are three possibilities, (1) the target is dead and therefore does not interact with the focal. This occurs with probability $1-l_{\tau}$. (2) The target is alive and interacts with the focal during period 1. This occurs with probability $l_{\tau}\left(1-q\right)$. (3) The target is alive and does not interact with the focal. This occurs with probability$l_{\tau}q$ . Thus the probability that they do not interact during the first period is

$$u_{1}=1-l_{\tau}+l_{\tau}q$$

Now we calculate the probability that they do not interact in either period 1 or period 2. We only have to worry about those pairs who were both alive and did not interact. Among those pairs there is a probability $1-\left(\frac{l_{2}}{l_{1}}\right)\left(\frac{l_{\tau+1}}{l_{\tau}}\right)$ that either the focal or the target dies between periods 1 and 2 given that they were both alive during period 1. The probability that they both survive, and don't interact during period 2 is $\left(\frac{l_{2}}{l_{1}}\right)\left(\frac{l_{\tau+1}}{l_{\tau}}\right)q$ . Thus the probability that they don't interact in periods 1 and 2,$u_{2}$, is

$$u_{2}=1-l_{\tau}+l_{\tau}q\left(1-\left(\frac{l_{2}}{l_{1}}\right)\left(\frac{l_{\tau+1}}{l_{\tau}}\right)+\left(\frac{l_{2}}{l_{1}}\right)\left(\frac{l_{\tau+1}}{l_{\tau}}\right)q\right)=1-l_{\tau}+q\left(l_{1}l_{\tau}-l_{2}l_{\tau+1}\right)+q^{2}l_{2}l_{\tau+1}$$

where the last term in the sum is the probability that both are alive during period 2 but have not interacted. Thus the probability that they do not interact during the first three periods is

$$\begin{matrix} u_{3}=1-l_{\tau}+q\left(l_{1}l_{\tau}-l_{2}l_{\tau+1}\right)+q^{2}l_{2}l_{\tau+1}\left(1-\left(\frac{l_{3}}{l_{2}}\right)\left(\frac{l_{\tau+2}}{l_{\tau+1}}\right)+\left(\frac{l_{3}}{l_{2}}\right)\left(\frac{l_{\tau+2}}{l_{\tau+1}}\right)\right) \\ =1-l_{\tau}+q\left(l_{1}l_{\tau}-l_{2}l_{\tau+1}\right)+q^{2}\left(l_{2}l_{\tau+1}+l_{3}l_{\tau+2}\right)+q^{3}l_{3}l_{\tau+2} \end{matrix}$$

Repeating this process yields until period *T*

$Q_{z}=u_{T+1}=1-l_{\tau}+\sum_{t=1}^{T-\tau} q^{t}\left(l_{t}l_{\tau+t-1}-l_{t+1}l_{\tau+t}\right)$ (eqn. S4)

because $l_{T+1}=0$. The probability that the focal interacts with the target at least once is

$P_{\tau}=1-Q_{\tau}=l_{\tau}-\sum_{t=1}^{T-\tau} q^{t}\left(l_{t}l_{\tau+t-1}-l_{t+1}l_{\tau+t}\right)$ (eqn. S5)

There are $n_{1}-1$ possible targets in the focal's own cohort, and $n_{1}$ in each of the cohorts of older individuals. Since the labels focal and target are arbitrary, the probability of interacting with a younger individual, and therefore the expected number of younger interactants, is equal the number of older interactants. Since the probabilities of interacting with each target are assumed to be independent, the focal interacts with *N* individuals on average during his lifetime where

$N=\left(n_{1}-1\right)P_{1}+2n_{1}\sum_{\tau=2}^{T} P_{\tau}$ (eqn. S6)

Notice here that *N* is roughly proportional to *n*_1_ when *n*_1_ becomes moderately large (eg. > 10). Adult survivorship for each population is shown in Table S15. Since the number of individuals in the first adult cohort (n_1_) directly determines the total number of adults in a stable population with constant survival probabilities, *N* can be expressed as a function of adult population size and the yearly probability of interaction. This result is shown in Figure 4 of the text. Above a yearly interaction rate of about 0.3 for the two hunter-gatherer populations, the lifetime number of expected adult interactants is approximately twice that of the stationary adult population size.

We also carried out a sensitivity analyses to see whether mean lifetime expected proportion of the population that are interactants changes if we allow heterogeneity in *P_i_* due to the effects of independent variables in Table 2. Monte Carlo simulations show only tiny differences (proportional differences ranging from 4x10^-5^ to 0.015 of the predicted value using the means for each independent variable in Table 3) between the average proportion of the population that an individual interacts with during his/her lifetime depending on whether *P_i_* is taken as the overall population average measure, or is allowed to vary in the simulation according to the independent variables in Table 2 of the text.

Since we have now calculated the proportion of the population that is expected to interact over a lifetime, we can multiply this by the standing population size of interactants (*N*) for male-male dyads of Ache, Hadza and wild chimpanzees. The number of total adult males in all cohorts was estimated to be 11 for wild chimpanzees [14], 169 for precontact Northern Ache, and 281 for current Eastern Hadza. Plugging the population sizes into equation 4, we can calculate the expected number of adult interactants in a lifetime. For example, chimpanzee males who interact with every other male in their community in a lifetime (1-*q* = *p* = 1) and live in mean community sizes of 11 males, can expect to interact with 21 other males in a lifetime (asymptotic proportion of interactants = 1.935, x mean community size of 11 adult males). [Because of unknown dispersal patterns we unable to compute this number for female chimpanzees.] In contrast, Ache men with a yearly interaction rate of only 0.05 can expect to engage in that interaction type with 177 other men in a lifetime (population proportion of interactants = 1.04, x mean population size of 169 adult males). Hadza men with a yearly interaction rate of 0.05 will expect to engage in that interaction type with 300 adult males in a lifetime (population proportion of interactants = 1.07 x mean population size of 281 adult males). The calculated number of expected lifetime interactants for Ache and Hadza men, for each different measured interaction type is shown in Table S16. They range from a low of 101 adult men who are expected to provide food when incapacitated during the lifetime of an Ache man, to 427 Hadza men who are expected to engage in a conversation sometime during the life of any Hadza man who reaches age 15.

The conclusion is quite striking. Male hunter-gatherers are expected to interact with 5-35 times as many partners as are male chimpanzees depending on interaction type. The number of interaction partners of female hunter-gatherers is similar to that of males although we are not sure about the interaction patterns of female chimpanzees, who unlike males, transfer in adolescence and show a more limited activity range within communities.

5. Cumulative culture

The calculations in the previous section suggest that adult Ache and Hadza hunter-gatherers will observe, in their lifetime, more than 300-400 same sex adult models manufacturing tools, whereas chimpanzee males would observe only about 21 other males in a lifetime. These estimates of cultural models that will be observed in a lifetime allow us, by adopting certain assumptions, to calculate the probability that cultural adaptedness will increase or decrease from the average from the previous cultural generation. Henrich [15] presents a simple model that posits a population level of cultural adaptedness with mean value *z_t_* at time *t*, and a presumed error rate during imitation (parameter *α* in his model) that generally leads to a deterioration of this level by imperfect imitators. When the distribution of skill and technology expressed by imitators shows a mean utility level of *z − α,* but the skill level of different social learners approximates a Gumbel distribution with dispersion parameter *β* due to random error and innovation, there is a small tail of individuals in the distribution who actually acheive a skill or technology level with adaptive utility at time *z_t_*_+1_ that is higher than the original *z*_t_. If those skilled individuals become the preferred models for social learning in the next generation, the overall population cultural adaptedness (ie. accumulation of cultural improvements) can increase. Henrich shows that the change in cultural adaptedness level, ∆*z*, in each generation of copiers can be calculated as:

$\Delta\bar{z}=-\alpha+\beta\left(\varepsilon+\ln\left(N\right)\right)$ (eqn. S7)

By plugging in hypothetical values of *α* and *β* we can estimate the probability of a social learner observing a model, during his/her lifetime, that has a higher cultural utility value than that of the population modal value in the previous generation (*z*_t-1_). If success-biased transmission favors copying the most skilled individual in the population, we can therefore assess whether success-biased copying is likely to improve the cultural skill level in the population through time, based on the number of models that will be observed in a lifetime (see Henrich *15*: Figure 3). As an example of this we chose *α/β* ratio of 5, representing intermediate levels of imitation error and intermediate innovative variation in the adaptive utility acquired through social learning. Under these conditions the model predicts that chimpanzees will show a rapid deterioration in skill levels whereas both Ache and Hadza hunter-gatherers would show increases in cultural adaptedness with each transmission generation (main text Figure 3). Skills and technologies with this level of imitation fidelity and innovation after copying could be maintained and improved via social learning in human societies but they could not be maintained in among chimpanzees, even if chimpanzees had cognitively equivalent abilities of imitation and innovation (ie. *α/β* is the same for both species).

References and Notes:

1. K. R. Hill, A. M. Hurtado, Cooperative breeding in South American hunter-gatherers. *Proc. Roy. Soc. B.*  **276**, 3863 (2009).

2. K. Hill, A. M. Hurtado, *Ache life history: the ecology and demography of a foraging people* (Aldine, New York, 1996).

3. K. Hawkes, K. Hill, J. O’Connell, Why Hunters Gather: Optimal Foraging and the Ache of Eastern Paraguay. *Am. Ethnol.* 9, 379-398 (1982).

4. A. M. Hurtado, K. Hawkes, K. Hill, H. Kaplan, Female Subsistence Strategies Among Ache Hunter-Gatherers of Eastern Paraguay. *Hum. Ecol.* 13, 1-28 (1985).

5. H. Kaplan, K. Hill, Food Sharing Among Ache Foragers; Tests of Explanatory Hypotheses. *Curr. Anthropol.* 26, 223-245 (1985).

6. S. Callegari-Jaques, K. Hill, A. M. Hurtado, L.T. Rodriguez, C.H.D. Bau, F.M. Salzano, Genetic clues about the origin of Ache hunter-gatherers of Paraguay. American *J. Hum. Biol.* 20, 735-737 (2008).

7. F. W. Marlowe, *The Hadza: Hunter-gatherers of Tanzania* (Univ. of Calif. Press, 2010).

8. L. Gliganic, Z. Jacobs, R. Roberts, M. Domínguez-Rodrigo, A. Mabulla, New ages for Middle and Later Stone Age deposits at Mumba rockshelter, Tanzania: Optically stimulated luminescence dating of quartz and feldspar grains. *J. Hum.Evol.* 62, 533-547 (2012).

9. S.A. Tishkoff, Sarah et. al., The genetic structure and history of Africans and African Americans. *Science* 324, 1035-1044 (2009).

10. A. Z. Mabulla, Hunting and foraging in the Eyasi Basin, Northern Tanzania: past, present and future prospects. African Archaeol. Rev. 24, 15-33 (2007).

11. B. Sands, The linguistic relationship between Hadza and Khoisan, in M.Schladt, Ed. *Language, identity and conceptualization among the Khoisan*. (Rudiger Kupper Verlag, Koln, Germany, p. 266–283, 1998).

12. K. R. Hill, et al. Co-residence patterns in hunter-gatherer societies show unique human social structure. *Science* 331, 1286–1289 (2011).

13. Peduzzi, P., Concato, J., Kemper, E., Holford, T. R., & Feinstein, a R. (1996). A simulation study of the number of events per variable in logistic regression analysis. *Journal of clinical epidemiology*, *49*(12), 1373–1379.

14. J. C. Mitani, Demographic influences on the behavior of chimpanzees, Primates, 47, 6-13 (2006).

15. J. Henrich, Demography and Cultural Evolution: How Adaptive Cultural Processes can Produce Maladaptive Losses: The Tasmanian Case. *Am. Antiq*. 69, 197-214 (2004).

16. K. Hill, C. Boesch, J. Goodall, A. Pussey, J. Williams, R. Wrangham, Chimpanzee Mortality in the Wild. *J. Hum. Evol.* 40, 437-450 (2001).

17. N. Blurton Jones, L.C. Smith, J. F. O’Connell, K. Hawkes, C. L. Kamuzora, Demography of the Hadza, an Increasing and High Density Population of Savanna Foragers, Amer. J. Phys. Anthro. 89, 159-181 (1992).


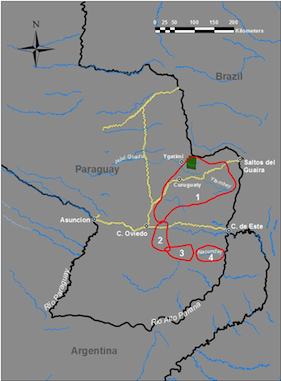


Fig. S1. The four dialect subtribes of Ache with their 20th century home ranges indicated (1- Northern; 2- Yvytyryzu; 3- Ypeti; 4- Ñacunday). The green square shows the modern day Mbarcayu reserve, where the last free forest living Ache were contacted in 1978.


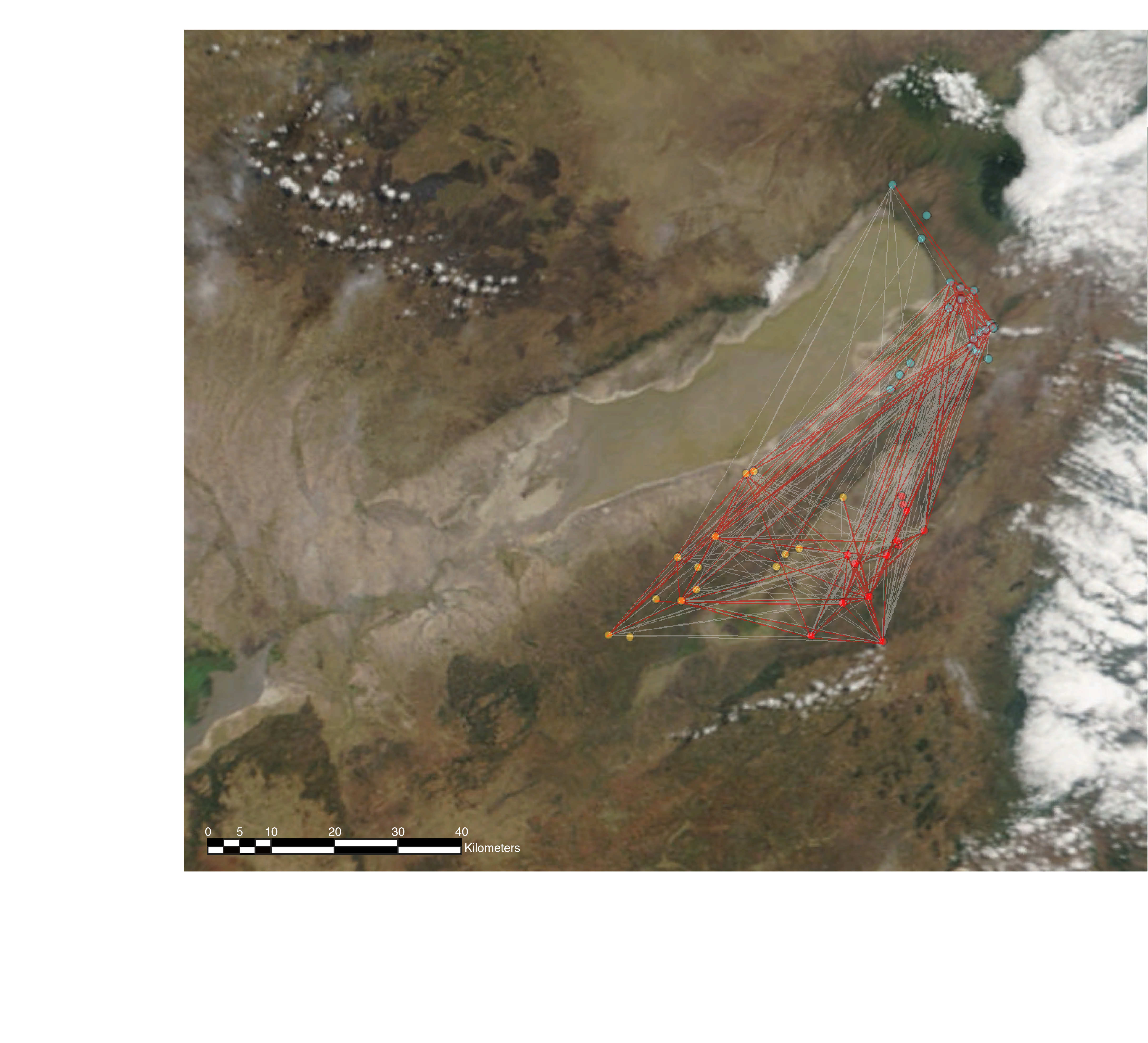


**Fig. S2.** Hadza camps included in the interviews. Each line connects the residential camp of all subject-target pairs at the time of the interview. Links drawn in red are cases in which subjects reported spending >100 days with a target over the 7 year reporting period, while dyads with less interaction are in light grey. Satellite image from NASA Earth Observatory.

**
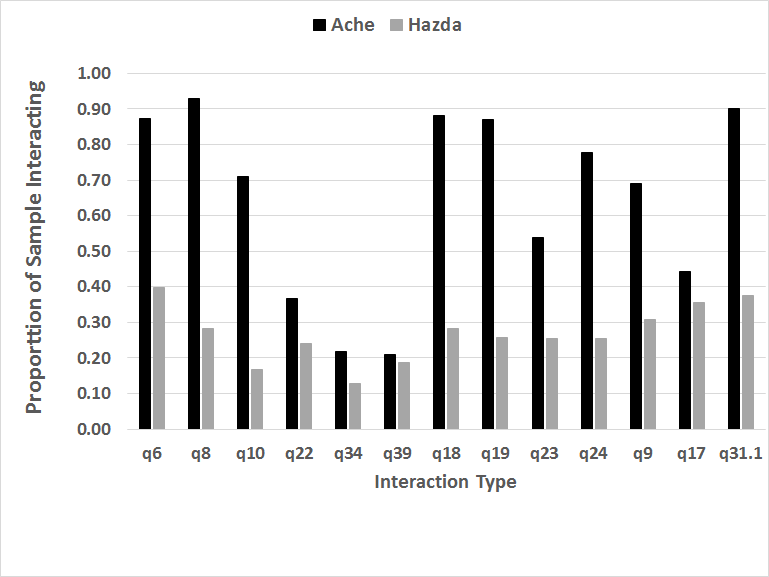
**

**Fig. S3.** Raw proportion of sample having a specific type of interaction for Ache and Hadza. The list of interaction types is provided in table S1.

**
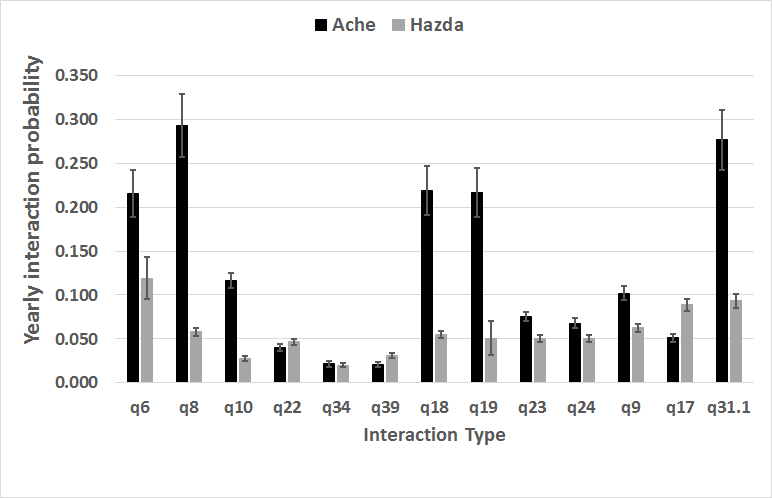
**

**Fig. S4.** Estimated yearly interaction rate and standard error (see methods) for Ache and Hadza adults. Interactions rates are based on the model with a constant term (b0) and a vector of three independent variables (*x_d_* ) comprising closekin, affinal kin, and ritual relationship at *t* = 1. Interaction types are defined in table S1.


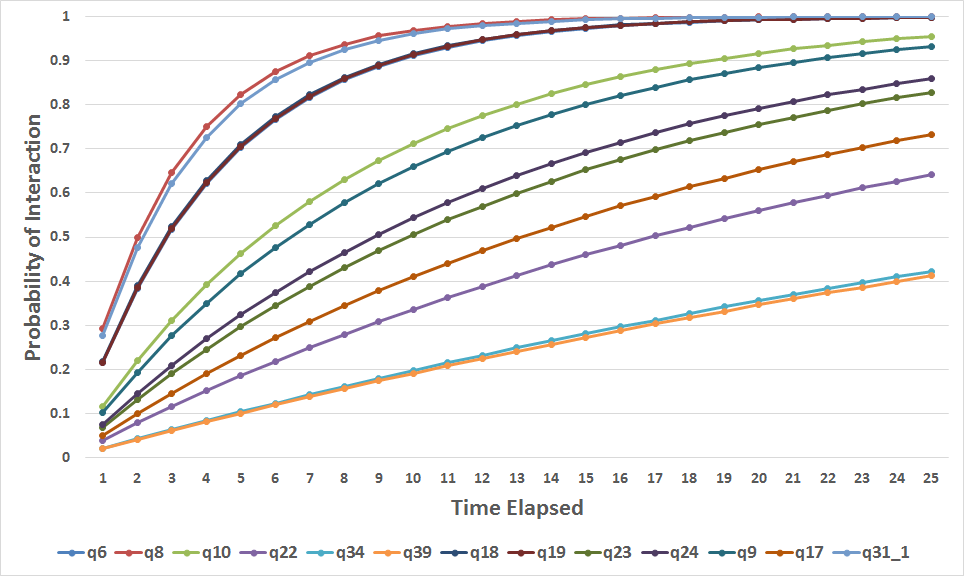


**Fig. S5.** Average probability of interaction between dyads per interaction type for Ache calculated from eqn S1 with control variables *x_d_* including genetic kinship, affinal kinship, and ritual relationship.


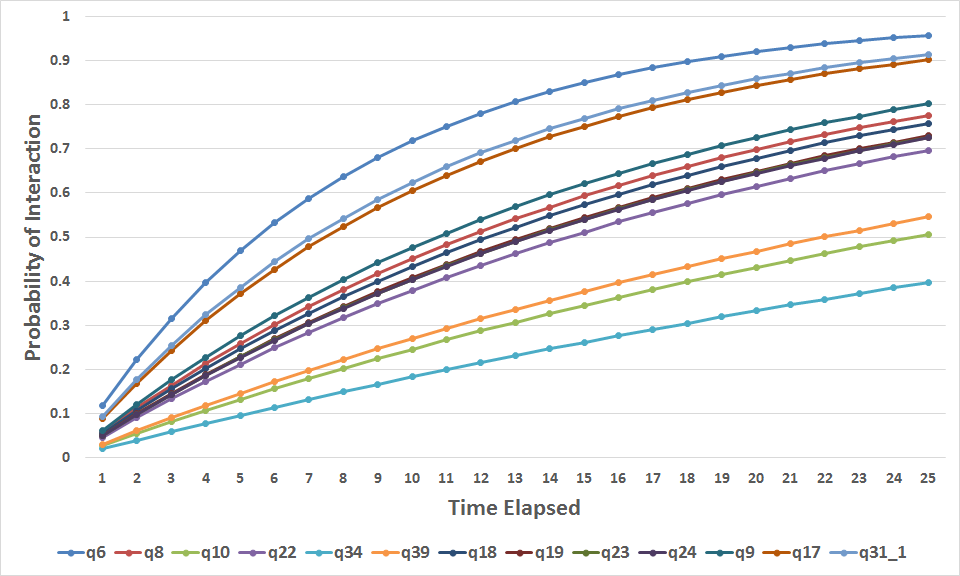


**Fig. S6.** Average probability of interaction between dyads per interaction type for Hazda calculated from eqn S1 with control variables *x_d_* being ritualual relationship, closekinship and affine.

**Table S1.** Reported interaction probabilities for same sex dyads for all questions about affiliative or cooperative interaction asked in the Ache and Hazda questionnaire. Numbers show the proportion of subjects reporting that “yes”, an interaction took place at least once during the specified time period.

| **Variable** | **Question to Interviewee:** | **Sample proportion interacting** | | | |
| --- | --- | --- | --- | --- | --- |
|  |  | **Ache**  **M-M** | **Ache**  **F-F** | **Hadza**  **M-M** | **Hadza**  **F-F** |
|  | **Associate** |  |  |  |  |
| q6 | Have you spoken with target? | 0.89 | 0.83 | 0.44 | 0.35 |
| q7 | Have you sat next to target? | 0.92 | 0.86 | na | na |
| q8 | Did target sleep in your camp? | 0.95 | 0.89 | 0.33 | 0.24 |
| q10 | Have you joked with target? | 0.76 | 0.58 | 0.19 | 0.14 |
| qH1 | Do you know target's name? | na | na | 0.72 | 0.60 |
|  | **Intimate Associate** |  |  |  |  |
| q11 | Did you sleep at target's fire hearth? | 0.45 | 0.39 | na | na |
| q13 | Have you bathed with target? | 0.69 | 0.52 | na | na |
| q15 | Have you tickled with target? | 0.58 | 0.38 | na | na |
| q22 | Has target given you a non food gift? | 0.38 | 0.34 | 0.26 | 0.23 |
| q34 | Did target ever groom you? | 0.19 | 0.29 | 0.11 | 0.14 |
| q35 | Did target ever paint you? | 0.06 | 0.12 | na | na |
| q36 | Did target ever shave you or cut your hair? | 0.05 | 0.13 | na | na |
|  | **Caretaker** |  |  |  |  |
| q5 | Did target help raise you (as a child)? | 0.28 | 0.28 | na | na |
| q38 | Did target medicate (plants, chants) you when you were sick or injured? | 0.23 | 0.12 | na | na |
| q39 | Did target give you food when you were sick or injured? | 0.24 | 0.14 | 0.14 | 0.23 |
| q40 | Did target ever care for your child? | 0.47 | 0.48 | na | na |
|  | **Supporter** |  |  |  |  |
| q32 | Did target ever defend you (verbally) when others were attacking you? | 0.53 | 0.61 | na | na |
| q33 | Did target ever defend you (side with you) in a physical (violent) confrontation? | 0.29 | 0.59 | na | na |
|  | **Cooperator** |  |  |  |  |
| q18 | Has target shared non meat foods with you? | 0.90 | 0.85 | 0.29 | 0.28 |
| q19 | Has target shared meat with you? | 0.89 | 0.82 | 0.30 | 0.22 |
| q21 | Did target ever request you to do something? | 0.50 | 0.28 | na | na |
| q23 | Did target ever lend you something? | 0.57 | 0.46 | 0.25 | 0.26 |
| q24 | Did you ever hunt/collect roots with target? | 0.78 | na | 0.22 | 0.30 |
| q25 | Did target ever wait for you while digging out armadillo? | 0.68 | na | na | na |
| q27 | Did target ever sharpen an arrow for you? | 0.23 | na | na | na |
| q28 | Did target ever call to you to cut a palm? | 0.49 | 0.13 | na | na |
| q29 | Did target ever carry your killed game back to camp? | 0.68 | na | na | na |
| q30 | Did target ever steal manioc with you? | 0.75 | 0.61 | na | na |
| q41 | Did target ever carry your child when walking a long distance? | 0.09 | 0.07 | na | na |
|  | **Cultural Model** |  |  |  |  |
| q9 | Have you heard target sing? | 0.67 | 0.73 | 0.33 | 0.28 |
| q14 | Have you constructed something with target (eg. honey scaffolding, bridge)? | 0.43 | na | na | na |
| q17 | Has target shared news with you? | 0.54 | 0.20 | 0.40 | 0.31 |
| q31 | Did target ever teach you anything? | na | na | 0.12 | 0.15 |
| q31.1 | Did you ever watch target make a tool? | 0.92 | 0.86 | 0.41 | 0.34 |
| q52 | Did target ever show you how to make something? | 0.07 | 0.08 | na | na |

Notes: na = not available; question was not asked.

**Table S2.** Independent Variables: percentage of yes respondent.

| **Indep**  **Var.** | **Question from which is derived** | **Percentage of sample** | | |
| --- | --- | --- | --- | --- |
|  |  | **tot** | **Ache** | **Hazda** |
| Closekin | Ego and target closely related (genetic coefficient r >= 0.125) | 5.86 | 9.40 | 4.47 |
| Ritual | Ego and target have ritual relationship | 17.07 | 20.23 | 15.76 |
| Affine | Ego employs affinal term for target (ie. spouse's sibs or parents) | 16.31 | 19.94 | 14.88 |
| Samecamp | Target was present in a camp with ego during  random camp censuses (Ache) or during interview (Hazda) | 8.19 | 18.52 | 4.14 |
| Elapsed | Mean years at risk of adult interaction with target  (during pre-contact forest period for Ache) | 8.17 | 11.16 | 7.00 |
| Sex | Female to Female dyadic relationship | 42.41 | 27.92 | 48.10 |
|  | Male to Male dyadic relationship | 57.59 | 72.08 | 51.90 |
| Ethnicity | Ache | 28.19 | na (100) | na(0) |
|  | Hazda | 71.81 | na(0) | na(100) |

**Table S3.** Spearman Correlation between independent variables. (* = correlation is significant at the 95% level)

|  | closekin | affine | ritual | samecamp | sex | time  elapsed |
| --- | --- | --- | --- | --- | --- | --- |
| closekin | 1 |  |  |  |  |  |
| affine | -0.071* | 1 |  |  |  |  |
| ritual | 0.095* | 0.129* | 1 |  |  |  |
| samecamp | 0.106* | 0.097* | 0.299* | 1 |  |  |
| sex | 0.019 | 0.114* | 0.106* | 0.054 | 1 |  |
| time elapsed | 0.102* | 0.070* | 0.094* | 0.227* | 0.112* | 1 |

**Table S4.** Ache Coefficient estimation and standard errors for interaction types related to Associate deriving from eqn S1

| **Associate** | | | | | | | | | | | | | | | |
| --- | --- | --- | --- | --- | --- | --- | --- | --- | --- | --- | --- | --- | --- | --- | --- |
|  | **q6** | | | | | **q8** | | | | | **q10** | | | | |
| closekin | 0.532 | 0.542 | 0.525 | 0.520 |  | 0.020 | 0.030 | 0.048 | 0.048 |  | -0.307 | -0.288 | -0.313 | -0.333 |  |
|  | (1.053) | (1.017) | (0.942) | (1.023) |  | (0.510) | (0.727) | (0.971) | (1.113) |  | (0.322) | (0.304) | (0.288) | (0.274) |  |
| affine | 0.017 | 0.015 | 0.031 |  |  | -0.001 | -0.003 | -0.003 |  |  | 0.460* | 0.432 | 0.409* |  |  |
|  | (0.338) | (0.302) | (0.268) |  |  | (0.333) | (0.365) | (0.351) |  |  | (0.227) | (0.221) | (0.205) |  |  |
| ritual | 0.738* | 0.724 | 0.413 | 0.414 |  | 0.280 | 0.237 | 0.358 | 0.357 |  | -0.043 | -0.035 | -0.114 | -0.055 |  |
|  | (0.374) | (0.378) | (0.433) | (0.466) |  | (0.410) | (0.457) | (0.469) | (0.483) |  | (0.245) | (0.225) | (0.204) | (0.199) |  |
| samecamp | -0.660* | -0.632* |  |  |  | 0.664 | 0.651 |  |  |  | -0.294 | -0.262 |  |  |  |
|  | (0.247) | (0.243) |  |  |  | (0.418) | (0.368) |  |  |  | (0.228) | (0.214) |  |  |  |
| sex | 0.226 |  |  |  |  | 0.174 |  |  |  |  | 0.437* |  |  |  |  |
|  | (0.192) |  |  |  |  | (0.217) |  |  |  |  | (0.185) |  |  |  |  |
| constant | -1.500* | -1.349* | -1.446* | -1.440* | -1.345* | -1.124* | -0.999* | -0.962* | -0.963* | -0.914* | -2.343* | -2.039* | -2.066* | -2.005* | -2.052* |
|  | (0.166) | (0.119) | (0.113) | (0.110) | (0.099) | (0.166) | (0.162) | (0.152) | (0.140) | (0.129) | (0.163) | (0.104) | (0.098) | (0.091) | (0.081) |
| N | 351 | 351 | 351 | 351 | 351 | 350 | 350 | 350 | 350 | 350 | 351 | 351 | 351 | 351 | 351 |
| ll | -110.059 | -110.748 | -114.325 | -114.334 | -116.780 | -68.120 | -68.380 | -69.428 | -69.428 | -70.015 | -200.855 | -204.275 | -205.113 | -207.286 | -208.273 |
| aic | 232.118 | 231.497 | 236.649 | 234.668 | 235.560 | 148.240 | 146.761 | 146.855 | 144.855 | 142.029 | 413.710 | 418.550 | 418.227 | 420.572 | 418.545 |
| McFaddenR^2^ | 0.058 | 0.052 | 0.021 | 0.021 | 0.000 | 0.027 | 0.023 | 0.008 | 0.008 | 0.000 | 0.036 | 0.019 | 0.015 | 0.005 | 0.000 |

Note: Bootstrapped standard errors in parenthesis. ll = log likelihood aic = Akaike information criterion. * = estimate significant at the 5% level. McFadden pseudo R^2^ is also reported and it is calculated as 1 – (ll_intercept_ / ll_full_) where ll_intercept_ = the log likelihood of the model without predictors and ll_full_ = log likelihood of the model with predictors.

**Table S5.** Ache Coefficient estimation and standard errors for interaction types related to Intimate Associate deriving from eqn S1

| **Intimate Associate** | | | | | | | | | | |
| --- | --- | --- | --- | --- | --- | --- | --- | --- | --- | --- |
|  | **q22** | | | | | **q34** | | | | |
| closekin | -0.085 | -0.082 | -0.131 | -0.097 |  | -0.320 | -0.274 | -0.298 | -0.268 |  |
|  | (0.341) | (0.341) | (0.342) | (0.333) |  | (1.053) | (0.469) | (0.820) | (0.436) |  |
| affine | -0.336 | -0.336 | -0.340 |  |  | -0.234 | -0.278 | -0.287 |  |  |
|  | (0.260) | (0.261) | (0.268) |  |  | (0.331) | (0.309) | (0.340) |  |  |
| ritual | 0.541* | 0.541* | 0.478* | 0.423 |  | 0.797* | 0.793* | 0.772* | 0.723* |  |
|  | (0.248) | (0.233) | (0.232) | (0.219) |  | (0.273) | (0.268) | (0.273) | (0.264) |  |
| samecamp | -0.257 | -0.258 |  |  |  | -0.092 | -0.104 |  |  |  |
|  | (0.274) | (0.268) |  |  |  | (0.305) | (0.307) |  |  |  |
| sex | -0.028 |  |  |  |  | -0.643* |  |  |  |  |
|  | (0.231) |  |  |  |  | (0.255) |  |  |  |  |
| constant | -3.160* | -3.181* | -3.211* | -3.268* | -3.178* | -3.478* | -3.920* | -3.931* | -3.980* | -3.812* |
|  | (0.212) | (0.137) | (0.125) | (0.113) | (0.096) | (0.219) | (0.170) | (0.159) | (0.154) | (0.120) |
| N | 350 | 350 | 350 | 350 | 350 | 350 | 350 | 350 | 350 | 350 |
| ll | -246.451 | -246.459 | -247.040 | -248.095 | -250.175 | -186.076 | -189.338 | -189.400 | -189.885 | -194.052 |
| aic | 504.901 | 502.919 | 502.080 | 502.190 | 502.350 | 384.152 | 388.677 | 386.799 | 385.770 | 390.103 |
| McFaddenR^2^ | 0.015 | 0.015 | 0.013 | 0.008 | 0.000 | 0.041 | 0.024 | 0.024 | 0.021 | 0.000 |

Note: Bootstrapped standard errors in parenthesis. ll = log likelihood aic = Akaike information criterion. * = estimate significant at the 5% level. McFadden pseudo R^2^ is also reported and it is calculated as 1 – (ll_intercept_ / ll_full_) where ll_intercept_ = the log likelihood of the model without predictors and ll_full_ = log likelihood of the model with predictors.

**Table S6.** Ache Coefficient estimation and standard errors for interaction types related to Caretaking deriving from eqn S1

| **Caretaker** | | | | | |
| --- | --- | --- | --- | --- | --- |
|  | **q39** | | | | |
| closekin | 0.455 | 0.391 | 0.406 | 0.372 |  |
|  | (0.404) | (0.401) | (0.388) | (0.388) |  |
| affine | 0.246 | 0.259 | 0.264 |  |  |
|  | (0.324) | (0.305) | (0.297) |  |  |
| ritual | 0.699* | 0.686* | 0.705* | 0.751* |  |
|  | (0.305) | (0.304) | (0.270) | (0.265) |  |
| samecamp | 0.051 | 0.065 |  |  |  |
|  | (0.307) | (0.320) |  |  |  |
| sex | 0.444 |  |  |  |  |
|  | (0.332) |  |  |  |  |
| constant | -4.491* | -4.142* | -4.135* | -4.082* | -3.837* |
|  | (0.328) | (0.175) | (0.158) | (0.159) | (0.122) |
| N | 350 | 350 | 350 | 350 | 350 |
| ll | -180.356 | -181.523 | -181.547 | -181.992 | -186.814 |
| aic | 372.712 | 373.046 | 371.094 | 369.985 | 375.629 |
| McFaddenR^2^ | 0.035 | 0.028 | 0.028 | 0.026 | 0.000 |

Note: Bootstrapped standard errors in parenthesis. ll = log likelihood aic = Akaike information criterion. * = estimate significant at the 5% level. McFadden pseudo R^2^ is also reported and it is calculated as 1 – (ll_intercept_ / ll_full_) where ll_intercept_ = the log likelihood of the model without predictors and ll_full_ = log likelihood of the model with predictors.

**Table S7.** Ache Coefficient estimation and standard errors for interaction types related to Cooperation deriving from eqn S1

| **Cooperator** | | | | | | | | | | |
| --- | --- | --- | --- | --- | --- | --- | --- | --- | --- | --- |
|  | **q18** | | | | | **q19** | | | | |
| closekin | -0.065 | -0.075 | -0.074 | -0.070 |  | -0.038 | -0.075 | -0.071 | -0.038 |  |
|  | (0.400) | (0.411) | (0.385) | (0.390) |  | (0.316) | (0.335) | (0.377) | (0.419) |  |
| affine | -0.019 | -0.023 | -0.026 |  |  | -0.201 | -0.201 | -0.210 |  |  |
|  | (0.303) | (0.295) | (0.302) |  |  | (0.277) | (0.274) | (0.259) |  |  |
| ritual | 0.888 | 0.875 | 0.888 | 0.882 |  | 0.976* | 0.912* | 0.962* | 0.913 |  |
|  | (0.488) | (0.485) | (0.457) | (0.497) |  | (0.482) | (0.450) | (0.474) | (0.477) |  |
| samecamp | 0.047 | 0.055 |  |  |  | 0.186 | 0.203 |  |  |  |
|  | (0.318) | (0.326) |  |  |  | (0.339) | (0.320) |  |  |  |
| sex | 0.048 |  |  |  |  | 0.232 |  |  |  |  |
|  | (0.201) |  |  |  |  | (0.200) |  |  |  |  |
| constant | -1.516* | -1.480* | -1.473* | -1.477* | -1.393* | -1.658* | -1.494* | -1.470* | -1.511* | -1.419* |
|  | (0.170) | (0.123) | (0.126) | (0.112) | (0.099) | (0.169) | (0.116) | (0.121) | (0.109) | (0.097) |
| N | 350 | 350 | 350 | 350 | 350 | 350 | 350 | 350 | 350 | 350 |
| ll | -126.715 | -126.746 | -126.770 | -126.777 | -131.342 | -124.766 | -125.523 | -125.824 | -126.288 | -131.205 |
| aic | 265.431 | 263.492 | 261.540 | 259.553 | 264.684 | 261.533 | 261.047 | 259.647 | 258.576 | 264.410 |
| McFaddenR^2^ | 0.035 | 0.035 | 0.035 | 0.035 | 0.000 | 0.049 | 0.043 | 0.041 | 0.037 | 0.000 |
| **Cooperator** | | | | | | | | | | |
|  | **q23** | | | | | **q24** | | | | |
| closekin | -0.249 | -0.261 | -0.178 | -0.139 |  | 0.038 | -0.209 | -0.216 | -0.201 |  |
|  | (0.304) | (0.301) | (0.285) | (0.284) |  | (0.487) | (0.290) | (0.281) | (0.289) |  |
| affine | -0.557* | -0.553* | -0.528* |  |  | -0.229 | -0.102 | -0.103 |  |  |
|  | (0.240) | (0.230) | (0.231) |  |  | (0.269) | (0.207) | (0.202) |  |  |
| ritual | 0.415 | 0.411* | 0.490* | 0.381 |  | 0.577 | 0.207 | 0.188 | 0.175 |  |
|  | (0.216) | (0.202) | (0.202) | (0.210) |  | (0.362) | (0.206) | (0.190) | (0.186) |  |
| samecamp | 0.434* | 0.438* |  |  |  | -0.313 | -0.064 |  |  |  |
|  | (0.235) | (0.230) |  |  |  | (0.282) | (0.219) |  |  |  |
| sex | 0.113 |  |  |  |  | 5.058 |  |  |  |  |
|  | (0.204) |  |  |  |  | (6.843) |  |  |  |  |
| constant | -2.768* | -2.683* | -2.626* | -2.715* | -2.646* | -6.945 | -2.499* | -2.507* | -2.527* | -2.511* |
|  | (0.187) | (0.109) | (0.109) | (0.100) | (0.084) | (6.854) | (0.108) | (0.104) | (0.093) | (0.083) |
| N | 349 | 349 | 349 | 349 | 349 | 351 | 351 | 351 | 351 | 351 |
| ll | -260.432 | -260.627 | -263.034 | -266.509 | -268.713 | -149.526 | -245.517 | -245.566 | -245.707 | -246.389 |
| aic | 532.864 | 531.253 | 534.068 | 539.018 | 539.425 | 311.051 | 501.033 | 499.131 | 497.413 | 494.778 |
| McFaddenR^2^ | 0.031 | 0.030 | 0.021 | 0.008 | 0.000 | 0.393 | 0.004 | 0.003 | 0.003 | 0.000 |

Note: Bootstrapped standard errors in parenthesis. ll = log likelihood aic = Akaike information criterion. * = estimate significant at the 5% level. McFadden pseudo R^2^ is also reported and it is calculated as 1 – (ll_intercept_ / ll_full_) where ll_intercept_ = the log likelihood of the model without predictors and ll_full_ = log likelihood of the model with predictors.

**Table S8.** Ache Coefficient estimation and standard errors for interaction types related to Cultural Model deriving from eqn S1

| **Cultural Model** | | | | | | | | | | | | | | | |
| --- | --- | --- | --- | --- | --- | --- | --- | --- | --- | --- | --- | --- | --- | --- | --- |
|  | **q9** | | | | | **q17** | | | | | **q31_1** | | | | |
| closekin | -0.411 | -0.359 | -0.414 | -0.393 |  | 0.238 | 0.143 | 0.174 | 0.202 |  | 0.084 | 0.049 | 0.045 | 0.024 |  |
|  | (0.287) | (0.290) | (0.294) | (0.287) |  | (0.305) | (0.294) | (0.297) | (0.293) |  | (0.946) | (1.137) | (0.967) | (1.104) |  |
| affine | -0.275 | -0.265 | -0.277 |  |  | -0.237 | -0.200 | -0.186 |  |  | -0.164 | -0.170 | -0.146 |  |  |
|  | (0.226) | (0.225) | (0.219) |  |  | (0.256) | (0.231) | (0.236) |  |  | (0.360) | (0.351) | (0.349) |  |  |
| ritual | 0.389 | 0.395 | 0.272 | 0.216 |  | 0.413 | 0.354 | 0.416* | 0.383 |  | 1.377* | 1.304* | 1.229 | 1.239 |  |
|  | (0.224) | (0.235) | (0.213) | (0.216) |  | (0.237) | (0.220) | (0.207) | (0.208) |  | (0.496) | (0.604) | (0.713) | (0.706) |  |
| samecamp | -0.375* | -0.406* |  |  |  | 0.133 | 0.216 |  |  |  | -0.356 | -0.330 |  |  |  |
|  | (0.228) | (0.227) |  |  |  | (0.250) | (0.233) |  |  |  | (0.426) | (0.519) |  |  |  |
| sex | -0.501* |  |  |  |  | 1.079* |  |  |  |  | 0.218 |  |  |  |  |
|  | (0.178) |  |  |  |  | (0.257) |  |  |  |  | (0.287) |  |  |  |  |
| constant | -1.714* | -2.096* | -2.147* | -2.195* | -2.193* | -3.868* | -3.018* | -2.993* | -3.028* | -2.921* | -1.325* | -1.180* | -1.231* | -1.261* | -1.191* |
|  | (0.155) | (0.113) | (0.106) | (0.099) | (0.081) | (0.248) | (0.119) | (0.118) | (0.108) | (0.091) | (0.197) | (0.171) | (0.158) | (0.148) | (0.133) |
| N | 351 | 351 | 351 | 351 | 351 | 350 | 350 | 350 | 350 | 350 | 244 | 244 | 244 | 244 | 244 |
| ll | -232.416 | -236.735 | -238.952 | -240.083 | -242.075 | -243.510 | -255.523 | -256.024 | -256.411 | -258.529 | -67.797 | -68.206 | -68.723 | -68.863 | -71.088 |
| aic | 476.833 | 483.469 | 485.905 | 486.166 | 486.150 | 499.021 | 521.045 | 520.048 | 518.823 | 519.057 | 147.593 | 146.412 | 145.446 | 143.726 | 144.175 |
| McFaddenR^2^ | 0.040 | 0.022 | 0.013 | 0.008 | 0.000 | 0.058 | 0.012 | 0.010 | 0.008 | 0.000 | 0.046 | 0.041 | 0.033 | 0.031 | 0.000 |

Note: Bootstrapped standard errors in parenthesis. ll = log likelihood aic = Akaike information criterion. * = estimate significant at the 5% level. McFadden pseudo R^2^ is also reported and it is calculated as 1 – (ll_intercept_ / ll_full_) where ll_intercept_ = the log likelihood of the model without predictors and ll_full_ = log likelihood of the model with predictors.

**Table S9.** Hazda Coefficient estimation and standard errors for interaction types related to Associate deriving from eqn S1

| **Associate** | | | | | | | | | | | | | | | |
| --- | --- | --- | --- | --- | --- | --- | --- | --- | --- | --- | --- | --- | --- | --- | --- |
|  | **q6** | | | | | **q8** | | | | | **q10** | | | | |
| closekin | 1.471* | 1.466* | 1.490* | 1.292* |  | 1.167* | 1.166* | 1.133* | 0.980* |  | 0.673 | 0.655 | 0.637 | 0.221 |  |
|  | (0.330) | (0.313) | (0.323) | (0.311) |  | (0.340) | (0.329) | (0.348) | (0.363) |  | (0.402) | (0.425) | (0.376) | (0.386) |  |
| affine | 1.273* | 1.264* | 1.308* |  |  | 0.837* | 0.835* | 0.834* |  |  | 1.487* | 1.454* | 1.444* |  |  |
|  | (0.184) | (0.180) | (0.169) |  |  | (0.215) | (0.210) | (0.218) |  |  | (0.217) | (0.224) | (0.213) |  |  |
| ritual | 2.821* | 2.801* | 2.862* | 2.741* |  | 2.056* | 2.051* | 2.193* | 2.280* |  | 1.599* | 1.562* | 1.671* | 1.871* |  |
|  | (0.568) | (0.554) | (0.695) | (0.602) |  | (0.185) | (0.184) | (0.176) | (0.165) |  | (0.224) | (0.229) | (0.219) | (0.181) |  |
| samecamp | 1.963* | 1.953* |  |  |  | 1.684 | 1.685 |  |  |  | 0.571 | 0.594 |  |  |  |
|  | (0.845) | (0.862) |  |  |  | (1.352) | (1.342) |  |  |  | (0.372) | (0.384) |  |  |  |
| sex | -0.063 |  |  |  |  | -0.019 |  |  |  |  | -0.166 |  |  |  |  |
|  | (0.145) |  |  |  |  | (0.163) |  |  |  |  | (0.205) |  |  |  |  |
| constant | -3.334* | -3.363* | -3.341* | -3.106* | -2.625* | -3.837* | -3.846* | -3.802* | -3.661* | -3.075* | -4.465* | -4.537* | -4.521* | -4.205* | -3.680* |
|  | (0.114) | (0.092) | (0.089) | (0.076) | (0.057) | (0.126) | (0.106) | (0.109) | (0.098) | (0.067) | (0.147) | (0.130) | (0.126) | (0.117) | (0.086) |
| N | 850 | 850 | 850 | 850 | 850 | 849 | 849 | 849 | 849 | 849 | 850 | 850 | 850 | 850 | 850 |
| ll | -397.466 | -397.558 | -404.060 | -428.939 | -567.302 | -362.937 | -362.944 | -374.679 | -384.698 | -495.951 | -296.722 | -297.087 | -298.903 | -324.055 | -373.719 |
| aic | 806.931 | 805.117 | 816.120 | 863.879 | 1136.604 | 737.875 | 735.888 | 757.357 | 775.395 | 993.902 | 605.444 | 604.173 | 605.805 | 654.110 | 749.439 |
| McFaddenR^2^ | 0.299 | 0.299 | 0.288 | 0.244 | 0.000 | 0.268 | 0.268 | 0.245 | 0.224 | 0.000 | 0.206 | 0.205 | 0.200 | 0.133 | 0.000 |

Note: Bootstrapped standard errors in parenthesis. ll = log likelihood aic = Akaike information criterion. * = estimate significant at the 5% level. McFadden pseudo R^2^ is also reported and it is calculated as 1 – (ll_intercept_ / ll_full_) where ll_intercept_ = the log likelihood of the model without predictors and ll_full_ = log likelihood of the model with predictors.

**Table S10.** Hazda Coefficient estimation and standard errors for interaction types related to Intimate Associate deriving from eqn S1

| **Intimate Associate** | | | | | | | | | | |
| --- | --- | --- | --- | --- | --- | --- | --- | --- | --- | --- |
|  | **q22** | | | | | **q34** | | | | |
| closekin | 1.172* | 1.141* | 1.098* | 0.987* |  | 1.206* | 1.089* | 1.053* | 1.035* |  |
|  | (0.332) | (0.349) | (0.364) | (0.354) |  | (0.354) | (0.353) | (0.367) | (0.349) |  |
| affine | 0.563* | 0.525* | 0.534* |  |  | 0.245 | 0.086 | 0.079 |  |  |
|  | (0.222) | (0.215) | (0.212) |  |  | (0.316) | (0.297) | (0.287) |  |  |
| ritual | 2.020* | 1.965* | 2.098* | 2.170* |  | 2.422* | 2.149* | 2.245* | 2.256* |  |
|  | (0.185) | (0.174) | (0.165) | (0.159) |  | (0.276) | (0.237) | (0.220) | (0.220) |  |
| samecamp | 0.904* | 0.982* |  |  |  | 0.296 | 0.535 |  |  |  |
|  | (0.391) | (0.444) |  |  |  | (0.406) | (0.354) |  |  |  |
| sex | -0.259 |  |  |  |  | -0.900* |  |  |  |  |
|  | (0.170) |  |  |  |  | (0.251) |  |  |  |  |
| constant | -3.833* | -3.949* | -3.926* | -3.841* | -3.237* | -4.529* | -4.851* | -4.837* | -4.825* | -4.015* |
|  | (0.134) | (0.108) | (0.104) | (0.099) | (0.071) | (0.170) | (0.161) | (0.160) | (0.162) | (0.099) |
| N | 850 | 850 | 850 | 850 | 850 | 850 | 850 | 850 | 850 | 850 |
| ll | -357.270 | -358.525 | -363.906 | -367.677 | -464.931 | -235.514 | -243.341 | -244.640 | -244.683 | -307.879 |
| aic | 726.540 | 727.051 | 735.812 | 741.354 | 931.862 | 483.027 | 496.682 | 497.280 | 495.365 | 617.758 |
| McFaddenR^2^ | 0.232 | 0.229 | 0.217 | 0.209 | 0.000 | 0.235 | 0.210 | 0.205 | 0.205 | 0.000 |

Note: Bootstrapped standard errors in parenthesis. ll = log likelihood aic = Akaike information criterion. * = estimate significant at the 5% level. McFadden pseudo R^2^ is also reported and it is calculated as 1 – (ll_intercept_ / ll_full_) where ll_intercept_ = the log likelihood of the model without predictors and ll_full_ = log likelihood of the model with predictors.

**Table S11.** Hazda Coefficient estimation and standard errors for interaction types related to Caretaker deriving from eqn S1

| **Caretaker** | | | | | |
| --- | --- | --- | --- | --- | --- |
|  | **q39** | | | | |
| closekin | 1.287* | 1.069* | 1.015* | 0.928* |  |
|  | (0.328) | (0.355) | (0.348) | (0.351) |  |
| affine | 0.617* | 0.375 | 0.368 |  |  |
|  | (0.259) | (0.241) | (0.227) |  |  |
| ritual | 2.246* | 1.860* | 1.988* | 2.040* |  |
|  | (0.225) | (0.201) | (0.190) | (0.187) |  |
| samecamp | 0.382 | 0.709* |  |  |  |
|  | (0.395) | (0.344) |  |  |  |
| sex | -1.248* |  |  |  |  |
|  | (0.211) |  |  |  |  |
| constant | -3.872* | -4.285* | -4.268* | -4.210* | -3.579* |
|  | (0.129) | (0.125) | (0.122) | (0.124) | (0.084) |
| N | 850 | 850 | 850 | 850 | 850 |
| ll | -297.913 | -318.856 | -321.689 | -323.096 | -394.555 |
| aic | 607.825 | 647.711 | 651.378 | 652.192 | 791.110 |
| McFaddenR^2^ | 0.245 | 0.192 | 0.185 | 0.181 | 0.000 |

Note: Bootstrapped standard errors in parenthesis. ll = log likelihood aic = Akaike information criterion. * = estimate significant at the 5% level. McFadden pseudo R^2^ is also reported and it is calculated as 1 – (ll_intercept_ / ll_full_) where ll_intercept_ = the log likelihood of the model without predictors and ll_full_ = log likelihood of the model with predictors.

**Table S12.** Hazda Coefficient estimation and standard errors for interaction types related to Cooperator deriving from eqn S1

| **Cooperator** | | | | | | | | | | |
| --- | --- | --- | --- | --- | --- | --- | --- | --- | --- | --- |
|  | **q18** | | | | | **q19** | | | | |
| closekin | 1.057* | 1.013* | 0.973* | 0.870* |  | 1.296* | 1.297* | 1.262* | 1.129* |  |
|  | (0.339) | (0.326) | (0.374) | (0.381) |  | (0.317) | (0.327) | (0.339) | (0.359) |  |
| affine | 0.617* | 0.556* | 0.566* |  |  | 0.738* | 0.742* | 0.709* |  |  |
|  | (0.214) | (0.207) | (0.208) |  |  | (0.201) | (0.204) | (0.216) |  |  |
| ritual | 2.056* | 1.951* | 2.087* | 2.161* |  | 2.001* | 2.005* | 2.133* | 2.218* |  |
|  | (0.182) | (0.178) | (0.163) | (0.157) |  | (0.187) | (0.179) | (0.167) | (0.156) |  |
| samecamp | 0.865 | 0.962 |  |  |  | 0.817* | 0.810* |  |  |  |
|  | (0.581) | (0.625) |  |  |  | (0.386) | (0.397) |  |  |  |
| sex | -0.376* |  |  |  |  | 0.021 |  |  |  |  |
|  | (0.163) |  |  |  |  | (0.158) |  |  |  |  |
| constant | -3.574* | -3.736* | -3.718* | -3.628* | -3.077* | -3.942* | -3.932* | -3.907* | -3.788* | -3.180* |
|  | (0.115) | (0.101) | (0.100) | (0.090) | (0.071) | (0.141) | (0.107) | (0.109) | (0.101) | (0.071) |
| N | 850 | 850 | 850 | 850 | 850 | 850 | 850 | 850 | 850 | 850 |
| ll | -381.841 | -384.693 | -389.829 | -394.488 | -496.267 | -361.896 | -361.905 | -365.656 | -372.591 | -476.330 |
| aic | 775.682 | 779.386 | 787.658 | 794.976 | 994.533 | 735.793 | 733.810 | 739.312 | 751.182 | 954.660 |
| McFaddenR^2^ | 0.231 | 0.225 | 0.214 | 0.205 | 0.000 | 0.240 | 0.240 | 0.232 | 0.218 | 0.000 |
| **Cooperator** | | | | | | | | | | |
|  | **q23** | | | | | **q24** | | | | |
| closekin | 1.196* | 1.149* | 1.122* | 0.999* |  | 0.926* | 0.881* | 0.843* | 0.701 |  |
|  | (0.298) | (0.287) | (0.297) | (0.325) |  | (0.429) | (0.366) | (0.384) | (0.382) |  |
| affine | 0.730* | 0.650* | 0.650* |  |  | 0.946* | 0.705* | 0.696* |  |  |
|  | (0.212) | (0.203) | (0.201) |  |  | (0.200) | (0.200) | (0.196) |  |  |
| ritual | 1.967* | 1.867* | 1.994* | 2.068* |  | 2.523* | 2.098* | 2.189* | 2.248* |  |
|  | (0.179) | (0.173) | (0.158) | (0.156) |  | (0.216) | (0.178) | (0.168) | (0.166) |  |
| samecamp | 0.637 | 0.785* |  |  |  | 0.376 | 0.624* |  |  |  |
|  | (0.398) | (0.465) |  |  |  | (0.423) | (0.337) |  |  |  |
| sex | -0.406* |  |  |  |  | -1.168* |  |  |  |  |
|  | (0.163) |  |  |  |  | (0.206) |  |  |  |  |
| constant | -3.653* | -3.826* | -3.810* | -3.702* | -3.152* | -3.508* | -3.918* | -3.901* | -3.782* | -3.185* |
|  | (0.127) | (0.105) | (0.102) | (0.093) | (0.069) | (0.118) | (0.107) | (0.108) | (0.103) | (0.073) |
| N | 850 | 850 | 850 | 850 | 850 | 850 | 850 | 850 | 850 | 850 |
| ll | -377.588 | -380.819 | -384.500 | -390.492 | -481.792 | -340.282 | -363.063 | -365.271 | -371.731 | -475.219 |
| aic | 767.176 | 771.638 | 776.999 | 786.984 | 965.583 | 692.564 | 736.125 | 738.542 | 749.461 | 952.437 |
| McFaddenR^2^ | 0.216 | 0.210 | 0.202 | 0.190 | 0.000 | 0.284 | 0.236 | 0.231 | 0.218 | 0.000 |

Note: Bootstrapped standard errors in parenthesis. ll = log likelihood aic = Akaike information criterion. * = estimate significant at the 5% level. McFadden pseudo R^2^ is also reported and it is calculated as 1 – (ll_intercept_ / ll_full_) where ll_intercept_ = the log likelihood of the model without predictors and ll_full_ = log likelihood of the model with predictors.

**Table S13.** Hazda Coefficient estimation and standard errors for interaction types related to Cultural Model deriving from eqn S1

| **Cultural Model** | | | | | | | | | | | | | | | |
| --- | --- | --- | --- | --- | --- | --- | --- | --- | --- | --- | --- | --- | --- | --- | --- |
|  | **q9** | | | | | **q17** | | | | | **q31_1** | | | | |
| closekin | 1.021* | 0.998* | 0.965* | 0.827* |  | 1.554* | 1.556* | 1.565* | 1.439* |  | 1.092* | 1.082* | 1.075* | 0.904* |  |
|  | (0.343) | (0.358) | (0.361) | (0.372) |  | (0.314) | (0.313) | (0.318) | (0.323) |  | (0.389) | (0.418) | (0.413) | (0.427) |  |
| affine | 0.895* | 0.863* | 0.850* |  |  | 0.924* | 0.929* | 0.935* |  |  | 1.183* | 1.164* | 1.176* |  |  |
|  | (0.194) | (0.194) | (0.192) |  |  | (0.214) | (0.210) | (0.218) |  |  | (0.192) | (0.201) | (0.197) |  |  |
| ritual | 1.911* | 1.878* | 1.988* | 2.063* |  | 2.187* | 2.192* | 2.299* | 2.398* |  | 2.316* | 2.293* | 2.358* | 2.347* |  |
|  | (0.182) | (0.176) | (0.164) | (0.162) |  | (0.213) | (0.217) | (0.207) | (0.176) |  | (0.230) | (0.224) | (0.209) | (0.180) |  |
| samecamp | 0.688 | 0.724 |  |  |  | 0.983 | 0.976 |  |  |  | 0.359 | 0.384 |  |  |  |
|  | (0.532) | (0.373) |  |  |  | (0.810) | (0.764) |  |  |  | (0.561) | (0.549) |  |  |  |
| sex | -0.166 |  |  |  |  | 0.028 |  |  |  |  | -0.105 |  |  |  |  |
|  | (0.151) |  |  |  |  | (0.152) |  |  |  |  | (0.145) |  |  |  |  |
| constant | -3.536* | -3.610* | -3.592* | -3.448* | -2.956* | -3.432* | -3.418* | -3.401* | -3.250* | -2.742* | -3.337* | -3.384* | -3.379* | -3.173* | -2.708* |
|  | (0.117) | (0.093) | (0.097) | (0.089) | (0.066) | (0.123) | (0.092) | (0.091) | (0.080) | (0.062) | (0.114) | (0.091) | (0.092) | (0.077) | (0.060) |
| N | 850 | 850 | 850 | 850 | 850 | 850 | 850 | 850 | 850 | 850 | 850 | 850 | 850 | 850 | 850 |
| ll | -406.151 | -406.786 | -409.790 | -421.288 | -518.385 | -408.347 | -408.366 | -412.215 | -426.135 | -552.465 | -414.314 | -414.593 | -415.302 | -436.811 | -557.109 |
| aic | 824.301 | 823.571 | 827.581 | 848.577 | 1038.769 | 828.693 | 826.732 | 832.431 | 858.271 | 1106.929 | 840.628 | 839.186 | 838.604 | 879.622 | 1116.217 |
| McFaddenR^2^ | 0.217 | 0.215 | 0.209 | 0.187 | 0.000 | 0.261 | 0.261 | 0.254 | 0.229 | 0.000 | 0.256 | 0.256 | 0.255 | 0.216 | 0.000 |

Note: Bootstrapped standard errors in parenthesis. ll = log likelihood aic = Akaike information criterion. * = estimate significant at the 5% level. McFadden pseudo R^2^ is also reported and it is calculated as 1 – (ll_intercept_ / ll_full_) where ll_intercept_ = the log likelihood of the model without predictors and ll_full_ = log likelihood of the model with predictors.

**Table S14.** Average interaction rates between dyads separated by sex

| **Dep  Var.** | **Question to Interviewee:** | **Proportion of  sample interacting^#^** | | | | **Yearly interaction  probability^##^** | | | |
| --- | --- | --- | --- | --- | --- | --- | --- | --- | --- |
|  |  | **Ache  M-M** | **Ache  F-F** | **Hazda  M-M** | **Hazda  F-F** | **Ache  M-M** | **Ache  F-F** | **Hazda  M-M** | **Hazda  F-F** |
|  | **Associate** |  |  |  |  |  |  |  |  |
| q6 | Have you spoken with target? | 0.89 | 0.83 | 0.44 | 0.35 | 0.214 | 0.219 | 0.149 | 0.087 |
| q8 | Did target sleep in your camp? | 0.95 | 0.89 | 0.33 | 0.24 | 0.292 | 0.295 | 0.071 | 0.044 |
| q10 | Have you joked with target? | 0.76 | 0.58 | 0.19 | 0.14 | 0.117 | 0.115 | 0.035 | 0.021 |
|  | **Intimate Associate** |  |  |  |  |  |  |  |  |
| q22 | Has target given you a non food gift? | 0.38 | 0.34 | 0.26 | 0.23 | 0.040 | 0.041 | 0.056 | 0.036 |
| q34 | Did target ever groom you? | 0.19 | 0.29 | 0.11 | 0.14 | 0.022 | 0.022 | 0.024 | 0.016 |
|  | **Caretaker** |  |  |  |  |  |  |  |  |
| q39 | Did target give you food when you were sick or injured? | 0.24 | 0.14 | 0.14 | 0.23 | 0.021 | 0.022 | 0.037 | 0.025 |
|  | **Cooperator** |  |  |  |  |  |  |  |  |
| q18 | Has target shared non meat foods with you? | 0.90 | 0.85 | 0.29 | 0.28 | 0.218 | 0.223 | 0.066 | 0.043 |
| q19 | Has target shared meat with you? | 0.89 | 0.82 | 0.30 | 0.22 | 0.215 | 0.221 | 0.062 | 0.039 |
| q23 | Did target ever lend you something? | 0.57 | 0.46 | 0.25 | 0.26 | 0.068 | 0.069 | 0.061 | 0.040 |
| q24 | Did you ever hunt/collect roots with target? | 0.78 | 0.01 | 0.22 | 0.30 | 0.075 | 0.076 | 0.061 | 0.038 |
|  | **Cultural Model** |  |  |  |  |  |  |  |  |
| q9 | Have you heard target sing? | 0.67 | 0.73 | 0.33 | 0.28 | 0.102 | 0.102 | 0.076 | 0.049 |
| q17 | Has target shared news with you? | 0.54 | 0.20 | 0.40 | 0.31 | 0.051 | 0.052 | 0.108 | 0.067 |
| q31.1 | Did you ever watch target make a tool? | 0.92 | 0.86 | 0.41 | 0.34 | 0.274 | 0.283 | 0.115 | 0.070 |

Note: # = data are raw proportion of sample interacting for every specific interaction type. ## = yearly probability of interaction based on the model proposed in eqn S1 where the control variable vector comprises, genetic kin, affinal kin, and ritual plus a constant term at t=1.

**Table S15.** Adult survival probabilities for chimpanzees, Hadza, and Ache.
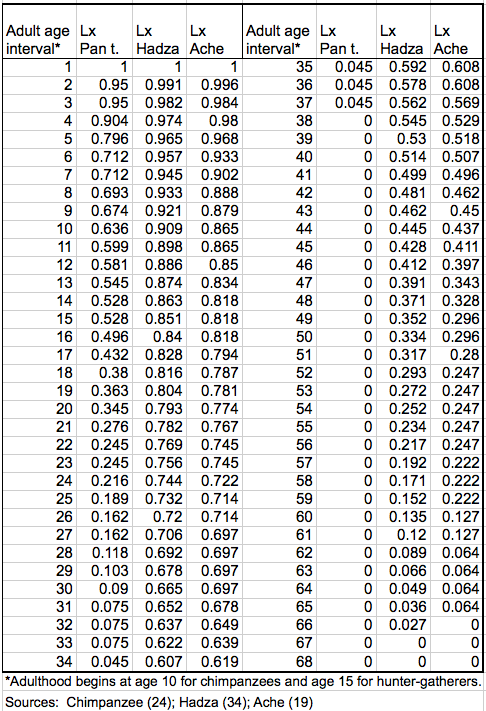


Sources: Chimpanzee [16]; Hadza [17]; Ache [2].

**Table S16.** Adult male expected lifetime interactants with measured mean values of p.

|  | **Question to Interviewee** | Yearly Probability of Interaction | | Lifetime Number of Interactants | |
| --- | --- | --- | --- | --- | --- |
|  | **Associate** | Ache | Hadza | Ache | Hadza |
| q6 | Have you spoken with target? | 0.215* | 0.12 | 290 | 427 |
| q8 | Did target sleep in your camp? | 0.293* | 0.06 | 304 | 324 |
| q10 | Have you joked with target? | 0.117* | 0.03 | 251 | 210 |
|  | **Intimate Associate** |  |  |  |  |
| q22 | Has target given you a non food gift? | 0.04 | 0.05 | 156 | 290 |
| q34 | Did target ever groom you? | 0.02 | 0.02 | 104 | 165 |
|  | **Caretaker** |  |  |  |  |
| q39 | Did target give you food when you were sick or injured? | 0.02 | 0.03 | 101 | 225 |
|  | **Cooperator** |  |  |  |  |
| q18 | Has target shared non meat foods with you? | 0.219* | 0.06 | 291 | 315 |
| q19 | Has target shared meat with you? | 0.217* | 0.05 | 290 | 303 |
| q23 | Did target ever lend you something? | 0.08 | 0.05 | 215 | 300 |
| q24 | Did you ever hunt/collect roots with target? | 0.07 | 0.05 | 206 | 303 |
|  | **Cultural Model** |  |  |  |  |
| q9 | Have you heard target sing? | 0.102* | 0.06 | 241 | 337 |
| q17 | Has target shared news with you? | 0.051* | 0.09 | 179 | 389 |
| q31.1 | Did you ever watch target make a tool? | 0.277* | 0.09 | 302 | 395 |
|  |  |  |  |  |  |
|  |  |  |  |  |  |
|  | Chimpanzee Lifetime number of interactants | 21 |  |  |  |
