# Supplementary material for: Hunter-Gatherer Inter-Band Interaction Rates: Implications for Cumulative Culture
Source: PLoS One. 2014 Jul 21;9(7):e102806. doi: 10.1371/journal.pone.0102806 (PMC4105570; doi:10.1371/journal.pone.0102806)
Supplement: DatabaseKey S1 — Key for Database. (DOCX) [file pone.0102806.s003.docx]

Ache_Hadza_Analyses_Dataset Key

Individual interview responses are in Row. 0 = no, 1 = yes

Column Item

1 ID of interview subject.

2 Ethnic group of interaction dyad.

3 Coefficient of genetic relatedness (r)between the subject and target of the dyad.

4 Is target close kin? [Is r from column 3 ≥ 0.125?]

5 Is target affinal kin? [Does spouse use a kin term for target, or do you use a kin term for target’s spouse?]

6 Is target a ritual partner? [Ache = Does target have a ritual relationship with you via your birth, target’s birth, the birth of your or target’s children, puberty ceremonies of you or target?] [ Hadza = Did you and target ever dance the Epeme dance together or eat Epeme meat together?]

7 Were subject and target ever censused in the same camp together? [Ache censuses come from 58 precontact random censuses done by recall] [Hadza = were subject and target living in the same camp on the interview day?]

8 Sex of interview dyad. [mm = male by male]

9 Number of years that the dyad was at risk of adult interaction. [Ache = years living in the forest up through 1971, and after both subject and target were ≥18 years old] [Hadza = 7 year period from time President was elected in Tanzania until interview date]

10 q6 Has subject ever spoken with target?

11 q8 Did target ever sleep in subject’s camp?

12 q10 Did subject ever joke with target?

13 q22 Did target ever give subject a non-food gift?

14 q34 Did target ever groom subject?

15 q39 Did target ever give food to subject when subject was sick or injured?

16 q19 Did target ever share meat with subject?

17 q18 Did target ever share non-meat food with subject?

18 q23 Did target ever lend something to subject?

19 q24 Did target ever hunt or collect plant foods with subject?

20 q9 Did subject ever hear target sing?

21 q17 Did target ever share news with subject?

22 q31_1 Did subject ever watch target make a tool?
